# The real-world evidence to the effects of primary psychological healthcare system in diluting risks of suicide ideation in underrepresented children/adolescents: an observational, multi-center, population-based, and longitudinal study

**DOI:** 10.1186/s13034-025-00914-4

**Published:** 2025-05-16

**Authors:** Wei Li, Xuerong Liu, Qianyu Zhang, Xiaobing Tian, Xianyong An, Jidong Ren, Xiaodi Han, Jingyu Lei, Chang Shen, Yanyan Li, Ji Chen, Lei Xia, Jingxuan Zhang, Yi Wu, Jie Gong, Hai Lan, Yan Wu, Zhengzhi Feng, Zhiyi Chen

**Affiliations:** 1 Experimental Research Center of Medical and Psychological Science (ERC-MPS), School of Psychology, Third Military Medical University, Chongqing, 400038 China; 2 Department of Public Management, Chongqing University, Chongqing, 400044 China; 3 Department of Epidemiology and Public Health Statistics, North Sichuan Medical College, Nanchong, 637000 Sichuan China; 4 Nanchong Psychosomatic Hospital (The Sixth People’s Hospital of Nanchong), Nanchong, 637000 Sichuan China; 5 Institute of Psychology and Behavioral Science, Shanghai Jiao Tong University, Shanghai, 200030 China; 6 Department of Psychology, Sichuan Normal University, Chengdu, 610068 Sichuan China; 7 School of Architecture, Zhengzhou University, Zhengzhou, 450001 Henan China; 8 Key Laboratory of Cognition and Personality, Ministry of Education, Faculty of Psychology, Southwest University, Chongqing, 400715 China

**Keywords:** Primary psychological healthcare system, Suicide ideation, Underprivileged children/adolescents

## Abstract

**Background:**

Establishing a primary psychological healthcare system to prevent suicide was eagerly advocated. Such system was developed as a low-cost healthcare framework integrating family, school, and hospitals to provide early psychological screening and intervention. However, it remains unclear whether such a policy-driven and low-cost healthcare system could be practical, especially with equal benefits for underrepresented children/adolescents. We aimed to examine the real-world practical effects of the primary psychological healthcare system in preventing suicide ideation among children/adolescents, particularly underprivileged ones.

**Methods:**

The study was conducted using an observational, multi-center, population-based, and longitudinal design. A total of 19,140 children and adolescents were sampled from lower- and middle-income areas in Nanchong, western China, with the majority for being underprivileged and underrepresented. They were followed up for one year. The primary outcome was the incidence of reported severe suicide ideation after implementing the primary psychological healthcare system at the 0.5-year and 1-year follow-ups, compared to baseline. Subgroup analysis was conducted to examine the equal benefits of the system for underrepresented children/adolescents.

**Results:**

The risks of suicide ideation for children/adolescents included in the system were found to be significantly lower compared to those not included at 0.5-year (adjusted relative risk [aRR] 0.28, 95%CI 0.23–0.33; *p* < 0.001) and 1-year follow-ups (aRR 0.28, 95% CI 0.23–0.33; *p* < 0.001). The effects were also observed among underrepresented children/adolescents, including “left-behind” children/adolescents, “single-parent” children/adolescents and children/adolescents in especially difficult circumstances (CEDC, all p_corrected_ < 0.001). The effects in “left-behind” children/adolescents, CEDC, and “single-parent” children/adolescents were found to be non-inferior to the typically developing cohort at non-inferiority thresholds of 30%, 35%, and 45%, respectively (all p_corrected_ < 0.05).

**Conclusions:**

The primary psychological healthcare system was effective in reducing suicide ideation risks among children/adolescents over a period of at least 1 year. However, certain underprivileged groups, such as orphans and unattended children, did not experience the same level of benefits, highlighting the need for targeted improvements.

**Supplementary Information:**

The online version contains supplementary material available at 10.1186/s13034-025-00914-4.

## Background

Death by suicide is sharply becoming one of the leading causes of increased mortality in children/adolescents around the world [1, 2]. According to reports from the United Nations International Children’s Emergency Fund (UNICEF) in 2021, 4 per 100,000 children/adolescents aged 10–19 died from suicide at home or even at school per year [3]. To make matters worse, approximately 80% of suicide deaths occurred in low- and middle-income countries (LMICs) [4]. In response to the suicide epidemic among children and adolescents, substantial financial investments (e.g., ¥1.1 billion under the “Healthy China Initiative 2023” policy) and ambitious policy reforms have been implemented, prioritizing the establishment of social psychological healthcare systems to enhance suicide prevention efforts [5–7]. Despite promising efforts and supports, the practices of these systems on preventing suicide in children/adolescents still confront challenges, as results of the fact that over 80% victims died from suicide despite having reached out to healthcare services [8]. Thus, it is imperative to explore practicable and effective psychological healthcare practice for suicide prevention, especially for children/adolescents.

As one of the promising solutions for suicide prevention, there is a growing call to integrate psychological services into primary healthcare systems to ensure universally essential psychological health services with equitable accesses aimed at controlling suicide [9–11]. Specifically, primary psychological healthcare system refers to a healthcare framework integrating families, schools, and hospitals [12, 13]. By providing early psychological screening and intervention for individuals at risk of psychological issues, and, when necessary, referring them to specialized psychiatric hospitals, it aims to ensure universally accessible psychological services and mental health care for all humans, especially vulnerable populations [12]. Despite remarkable efforts made to establish and practice primary psychological healthcare systems, there still exists a worldwide failure to provide mental health services to those in need, due to the global shortfall in mental health investment [14]. Over 70% of total mental health expenditures in middle-income countries are allocated to for-profit hospitals/institutions, far exceeding the investment in non-profit primary psychological healthcare systems, making non-profit systems operate in a low-cost manner [15]. Even more worryingly, the effectiveness of these non-profit systems is still questionable, especially in lower-middle-income regions of the Western Pacific, because operating with such low costs typically does not encourage high-quality services, leading to limited infrastructure, a shortage of human resources and limited community awareness of psychological health [16]. In addition to the “low-quality” concern, primary psychological healthcare systems still need to address potential inaccessibility and unavailability for underprivileged children/adolescents [3]. Given that children/adolescents are under development for independent self-care abilities, their help-seeking intentions and actions are usually arrested by their parents/caregivers due to poor mental health literacy and suicide stigma, especially in Chinese cultures [17, 18]. Considering these challenges, it is necessary to provide real-world evidence clarifying the practical effects of such system as a policy for suicide prevention among children/adolescents.

Compared to the primary physical healthcare system, the representation of children/adolescents remains disproportionately negligible in the primary psychological healthcare system, despite the principle of equitable distribution in such system, as highlighted in previous research [19]. The shortage of essential psychological services may handicap these children/adolescents in need from help-seeking [3]. Moreover, stigma associated with mental health problems functions as a substantial societal and cultural barrier to the practices of such systems among children/adolescents, especially in lower-middle-income areas. Poor literacy (e.g., mental health knowledge and education) leaves parents or even patients themselves to view the mental problems as immoral and disgraceful “dirty disorder” [20, 21]. To make matters worse, underprivileged children/adolescents have long been underrepresented in the healthcare system. Compared with typically developing children/adolescents raised in stable and supportive family environments, underprivileged individuals living in disadvantaged socio-cultural conditions (e.g., orphans, de facto unattended children/adolescents, “left-behind” children/adolescents, “single-parent” children/adolescents, and children/adolescents in especially difficult circumstances, as defined in Supplementary Methods 1) experience greater psychiatric burdens and suicidal risks [22], but are more likely to “drop out” of primary psychological healthcare systems, because such systems typically require high financial expenditure and the availability of these services is mostly limited to economically developed urban areas [19, 23]. Moreover, the social isolation and economic pressure caused by the COVID-19 pandemic may have exacerbated such disparities. During the pandemic, underprivileged individuals are more likely to experience mental health problems [24]. However, due to a lack of social support, these groups may face multiple barriers to accessing psychological services, including issues related to availability and their own willingness to seek help [25]. These challenges may further lead to the worsening of psychological distress, making their mental health status even more fragile. Therefore, we established a low-cost primary psychological healthcare system and aimed to provide real-world evidence to show whether underprivileged children/adolescents in LMICs equally benefit from psychological healthcare in a low-cost manner, especially during the COVID-19 pandemic.

In June 2022, during the ongoing challenges posed by the COVID-19 pandemic, Nanchong, one of the largest lower and middle-economic-status cities in western China, where over 60% of children/adolescents live in underprivileged conditions, implemented a citywide primary psychological healthcare system (the Psychological Health Guard for Children and Adolescents Project of China, CPHG) for all eligible children/adolescents, particularly those from underprivileged backgrounds. In light of this significant intervention, the primary research goal in the current study was to investigate the real-world effects of implementing this primary psychological healthcare system on preventing suicidal ideation among children/adolescents aged 10–19 in Nanchong.

## Methods

### Study design and participants

The study was a citywide, observational, multi-center, population-based, and longitudinal cohort study conducted in Nanchong, Sichuan Province, China, aiming to investigate the real-world effects of implementing the primary psychological healthcare system (CPHG) on controlling the risks of suicidal ideation, in which the exposure factor was whether participant was included in this system. Nanchong is a representative lower-middle-economic-status city in the western China (National Bureau of Statistics of P.R.C., 2022).

We firstly established 385 healthcare centers in each middle and high school, as well as in social welfare institutions in Nanchong. All children/adolescents who reported severe suicide ideation in the past two weeks at baseline were not included in the final analysis; however, they received immediate psychological services, clinical assessment, and medical treatment when necessary. At baseline, we included eligible participants who met the following criteria: First, informed consent was obtained from their parents or legal guardians. Individuals who refused to participate were excluded from this study. Second, individuals were assessed and identified as having no severe suicidal ideation at baseline. Only those who met both criteria were included in the study and further classified using the Center for Epidemiological Studies-Depression Scale (CES-D) to evaluate their risk of developing suicidal ideation in the future. Individuals with CES-D scores of 16 or higher were classified as “high risk”, while those with scores below 16 were classified as “low risk”. They received different interventions. However, it should be noted that during the study period, the local area was experiencing the COVID-19 pandemic and lockdown policies, which somewhat limited our tracking process and made it difficult to reach all participants, especially those in underprivileged groups, as well as contributed to unforeseen data loss.

Based on the locally legal definition of underprivileged conditions (Ministry of Civil Affairs of the PRC, 2019), the enrolled children/adolescents in the present study were further categorized into five underprivileged cohorts: de facto unattended children/adolescents, “left-behind” children/adolescents, “single-parent” children/adolescents, children/adolescents in especially difficult circumstance (CEDC), and orphan (Supplementary methods 1), as well as grouped for typically developing children/adolescents who were free from above conditions.

This study is reported in accordance with the Strengthening the Reporting of Observational Studies in Epidemiology (STROBE) guideline (Supplementary results 6).

### Procedure

The CPHG system adopted multiple projects to ensure its implementation, including the “2 + 2” psychological healthcare practice, psychological healthcare education, psychological healthcare training and psychological healthcare management. The details of these projects are provided in Supporting Document 2 (in Chinese) and Supporting Document 3 (translated into English). The key project was the “2 + 2” psychological healthcare practice. The former “2” represented two rounds of psychological screenings to capture the suicide ideation. In the first round, depression was defined as the risk factor, considering it was found to be one of the most common emotional disorders leading to suicide and ranks first in terms of disease burden in China [6]. The Center for Epidemiological Studies-Depression Scale (CES-D), widely used in epidemiological surveys, was employed for initial screening in this round [26]. Individuals with CES-D scores below 16 were identified as “low risk”, while those with CES-D scores of 16 or higher were identified as “high risk”. Once individuals identified as “high risk”, they underwent screening to measure suicide ideation with a single signaling question (i.e., have you ever felt hopeless for the future, giving rise to the idea of suicide? ) in the second round. Those identified with severe suicide ideation received two rounds of specific psychological care (interviews, the latter “2”). The first round of primary psychological care was conducted by trained and qualified staff at healthcare centers following structural guidelines. Those still identified “high risk” for suicide ideation after primary psychological care were transferred to the second-round preclinical (early) psychological interventions conducted by clinicians at government-sponsored hospitals (Supplementary methods 3). Screening for depressive symptoms and suicide ideation was implemented via purpose-built online software on cellphones called *Psychological Health Guard Project*. Meantime, healthcare centers lacking such infrastructure were supplemented by offline pencil-and-paper questionnaires. However, the data collected from traditional offline pencil-and-paper questionnaires were all unpredictably lost in the COVID-19 lockdown policy in China. Therefore, the data analyzed in the current study was all collected from purpose-built online software. Beyond the “2 + 2” psychological healthcare practices, the “psychological healthcare education” involved the dissemination of mental health knowledge through various formats (e.g., cartoons, videos, and animations). The “psychological healthcare training” included mental health training for healthcare staff, administrative leaders, head teachers, subject teachers, and full- or part-time mental health teachers and adopted a hierarchical approach, with different levels of staff receiving training content of varying depth. The comprehensive training curriculum and the requisite certification standards for the successful attainment of training completion are delineated within Supporting Document 3. The “psychological healthcare management” refers to a supportive framework to ensure the operation of the system, including mental health records management, psychological crisis prediction and early warning, and data curation (Supplementary document 2-technical book). At baseline, “high risk” children/adolescents were included in the primary psychological healthcare system, whereas “low risk” individuals (CES-D scores < 16) were not included in this system and were instead provided with routine social care.

### Outcome

The primary outcome was the incidence of severe suicide ideation reported by children/adolescents included in the CPHG (“high risk” individuals) at follow-up visits, compared to those outside the CPHG (“low risk” individuals).

### Statistical analysis

Descriptive statistical analyses were conducted using R (version 4.3.1), SPSS (IBM, Inc., version 29.0.1.0) and SPSSAU. The frequency (rate) was calculated with 95% confidence interval (CI, estimated by Bootstrapping method at *n* = 5000) to quantify the incidence of reporting suicide ideation.

As for the inferential statistics, the generalized linear mixed-effect models (GLMM) were built by *lmerTest* package in R, to test the effect size of implementing primary mental healthcare system on preventing suicide ideation [27]. A random effect for clustering of children/adolescents within regions was accounted to capture variability between groups. For missing data, we used different approaches depending on the variable’s role. Specifically, given that sociodemographic variables served primarily as covariates and had low levels of missingness, we applied dummy coding to retain the full sample and maintain statistical power. However, incomplete data in the CES-D (i.e., central to defining risk at suicide ideation) and suicide ideation (i.e., main outcome) measures could critically bias key outcomes. Therefore, participants lacking complete data on these measures were excluded to preserve the integrity of our primary analyses. For singular fit occurred in GLMM, we performed generalized linear model (GLM) to simplify the analysis. The GLMM for outcome included fixed effects for children/adolescents included or not included in the system, including comparison of the whole group and comparison of the subgroups (e.g., CEDC, orphan, de facto unattended children/adolescents, “single-parent” children/adolescents, and “left-behind children/adolescents”), adjusted for age, sex, offspring, family background (excluded in the comparison of subgroups) and depressive symptoms. By building upon the logit regression model, we calculated crude and adjusted relative risk (RR) and 95% CI for individuals included in the system compared with those outside the system at 0.5-year and 1-year follow-ups, respectively. Relative risk reduction (RRR) for such changes was also calculated to quantify the effectiveness of the CPHG.

Additionally, we also conducted a sensitivity analysis to examine the robustness. Specifically, we employed multiple imputation to address the missing data on suicide ideation in two follow-up assessments. Through multiple imputation, we generated a total of 5 imputed datasets. Subsequently, we generalized linear mixed models (GLMM) on each of these datasets, to minimize the potential bias arising from missing data and to ensure the robustness of results.

To further examine whether such benefits are equal effects in underprivileged children/adolescents, we used non-inferiority tests implemented by SPSSAU. Given that no evidence-based non-inferiority boundary values were provided previously, we tentatively set this liberal boundary value to range from 30 to 50% of the incidence rate in typically developing individuals, adjusting it in 5% increments. We estimated the sample size by using normal approximation method, with a two-sided α of 0.05, a β of 0.10. Holm-Bonferroni correction was employed to adjust p values for the multiple comparisons.

## Results

**Fig. 1:**
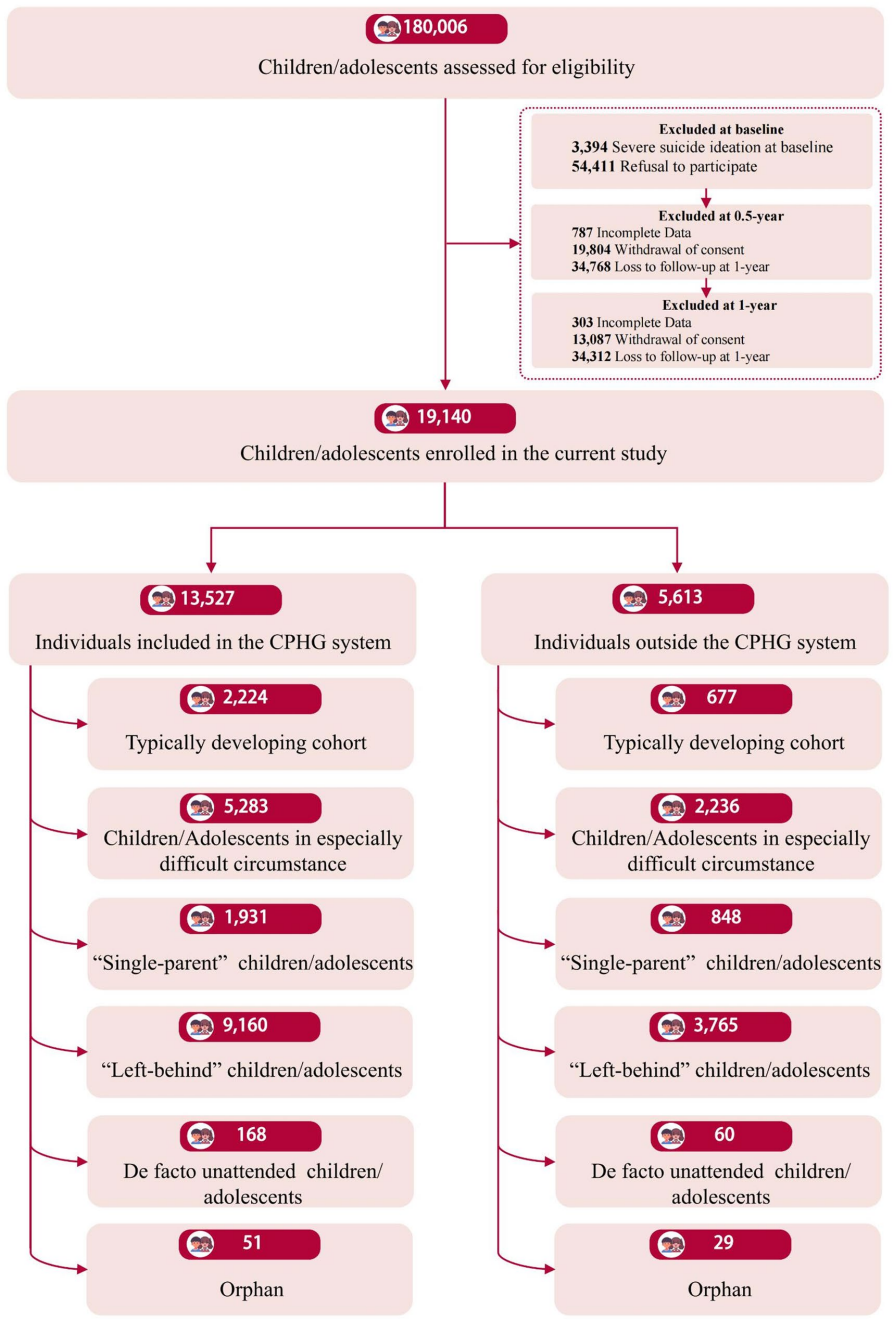
Screening and primary outcome population. The final cohorts for both included and excluded in CPHG system were categorized into five underprivileged conditions and one typically develop cohort. The criterion could be found in Supplementary methods 1

A total of 180,006 children/adolescents geographically representing the entire Nanchong were potentially screened in this study. However, due to the lockdown policies stemming from the COVID-19 pandemic and the concerns of guardians regarding suicide-related screening, many participants either declined to participate at the beginning (*N* = 54,411), withdrew their consent during the study (*N* = 32,891), or were lost to follow-up (*N* = 69,081). Additionally, 3,394 individuals had severe suicide ideation at the onset of the study, and 787 had missing data (128 in CES-D, 659 in suicide ideation) at the 0.5-year follow-up, while 303 had missing data in suicide ideation at the 1-year follow-up. Ultimately, only 19,140 eligible children/adolescents from 326 centers were included, with 13,527 in the CPHG system, and 5613 outside it (Fig. 1). Demographic characteristics have been tabulated (Table 1). Details for the subgroups (i.e., underprivileged cohorts) can be found in Table 2. Furthermore, to ensure homogeneity between individuals not included in the study and those included, particularly regarding the distribution of underprivileged individuals, we conducted differential analyses. Given the substantial difference in sample size between the excluded and the included individuals, even minor differences may result in significant statistical differences. Therefore, we also used effect sizes to evaluate the magnitude of these differences (Cramer’s V for categorical variables, Cohen’s d for continuous variables; Supplemental results 2). As shown in Table S6, although excluded and included individuals showed significant differences in all demographic variables, the effect sizes were small, suggesting that the differences in sample distribution may not possess practical significance, indicating that the attrition of the sample is not primarily sourced from children from underprivileged backgrounds or specific categories of other demographic variables (e.g., girls) and may not be sufficient to affect the study conclusions.

**Table 1:** Sociodemographic characteristics of the population enrolled in this study

	Children/adolescents included in the CPHG system (*n* = 13527)		Children/adolescents outside the CPHG system (*n* = 5613)		Total (*n* = 19140)	
	*n*	%	*n*	%	*n*	%
*Age*	14.22	1.50 (SD)	13.85 (1.48)	1.48 (SD)	14.11	1.51 (SD)
10–11	75	0.6%	62	1.1%	137	0.7%
12–14	7696	56.9%	3673	65.4%	11,369	59.4%
15–17	5629	41.6%	1854	33.0%	7483	39.1%
18–19	127	0.9%	24	0.4%	151	0.8%
*Sex*						
Boy	5146	38.0%	2216	39.5%	7362	38.5%
Girl	8381	62.0%	3397	60.5%	11,778	61.5%
*Offspring*						
Non-single child	11,259	83.2%	4645	82.8%	15,904	83.1%
Single child	2268	16.8%	968	17.2%	3236	16.9%
*Family background*						
Typically developing cohort	2224	16.4%	677	12.1%	2901	15.2%
Underprivileged cohort	11,210	82.9%	4864	86.7%	16,074	84.0%
Missing	93	0.8%	72	1.3%	165	0.9%
*CES-D*	24.40	8.01 (SD)	7.25	5.03 (SD)	19.37	10.67 (SD)

Data are mean (SD) or N (%). Missing = missing value. CES-D = Center for epidemiological survey, depression scale. Data were extracted from CPHG group across 596 sites covering almost all the areas of Nanchong City (Sichuan, China). A total of 180,006 children/adolescents were included, whereas 19,140 individuals were finally enrolled in the current study because of completely missing records, lack of informed consent, and loss to follow up. Underprivileged cohort consisted of orphan, de facto unattended children/adolescents, children/adolescents in especially difficult circumstance, “left-behind” children/adolescents, and “single-parent” children/adolescents

**Table 2:** Subgroup analysis of the practical effects of implementing primary psychological healthcare system

Cohorts	*N*	0.5-year follow-up					1-year follow-up				
		Case	%	aRR^a^	95% CI	*P* value	Case	%	aRR	95% CI	*P* value
*Typically developing cohort*											
Included^b^	2224	129	5.8%	0.39	0.24–0.65	< 0.001	118	5.3%	0.35	0.21–0.58	< 0.001
Outside^c^	677	44	6.5%	Reference			53	7.8%	Reference		
*Children/Adolescents in especially difficult circumstance*											
Included	5283	288	5.5%	0.28	0.21–0.37	< 0.001	302	5.7%	0.31	0.23–0.41	< 0.001
Outside	2236	179	8.0%	Reference			189	8.5%	Reference		
*“Single-parent” children/adolescents*											
Included	1931	141	7.3%	0.24	0.16–0.38	< 0.001	115	6.0%	0.28	0.18–0.44	< 0.001
Outside	848	90	10.6%	Reference			92	10.8%	Reference		
*“Left-behind” children/adolescents*											
Included	9160	534	5.8	0.26	0.21–0.33	< 0.001	511	5.6%	0.25	0.20–0.31	< 0.001
Outside	3765	296	7.9%	Reference			334	8.9%	Reference		
*De facto unattended children/adolescents*											
Included	168	15	8.9%	0.13	0.04–0.45	0.001	16	9.5%	0.32	0.09–1.19	0.089
Outside	60	11	18.3%	Reference			7	11.7%	Reference		
*Orphan*											
Included	51	7	13.7%	0.20	0.03–1.34	0.097	7	13.7%	0.55	0.08–3.80	0.542
Outside	29	8	27.6%	Reference			7	24.1%	Reference		

Generalized linear mixed models were used for the analysis. (a) adjusted relative risk, adjusted for all sociodemographic characteristics (age, sex, and offspring) and depression estimated by center for epidemiological survey, depression scale (CES-D). (b) included refers to children/adolescents included in primary psychological healthcare system. (c) outside refers to children/adolescents not included in primary psychological healthcare system

**Fig. 2:**
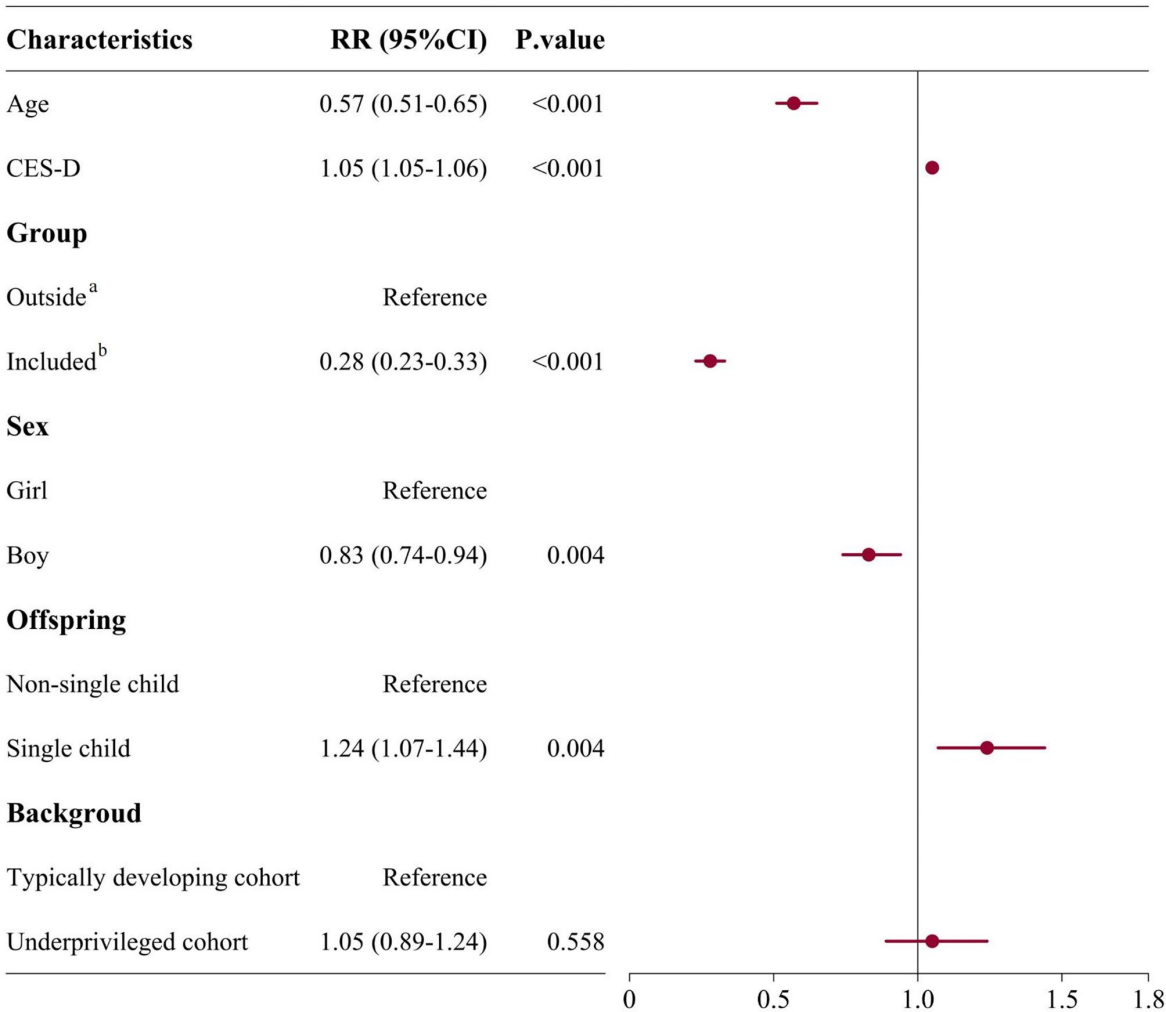
The adjusted Relative Risk (aRR) for the self-reporting suicide ideation at 0.5-year follow-up. **a***Outside* refers to children/adolescents not included in primary psychological healthcare system. **b***Included* refers to children/adolescents included in primary psychological healthcare system. The RRs were calculated by generalized linear mixed model. 95%CI = 95% Confidence Interval. CES-D = Center for Epidemiological Survey, Depression scale. When RR < 1, the variable is considered as protective factor for preventing suicide ideation. When RR > 1, the variable is considered as risk factor

**Fig. 3:**
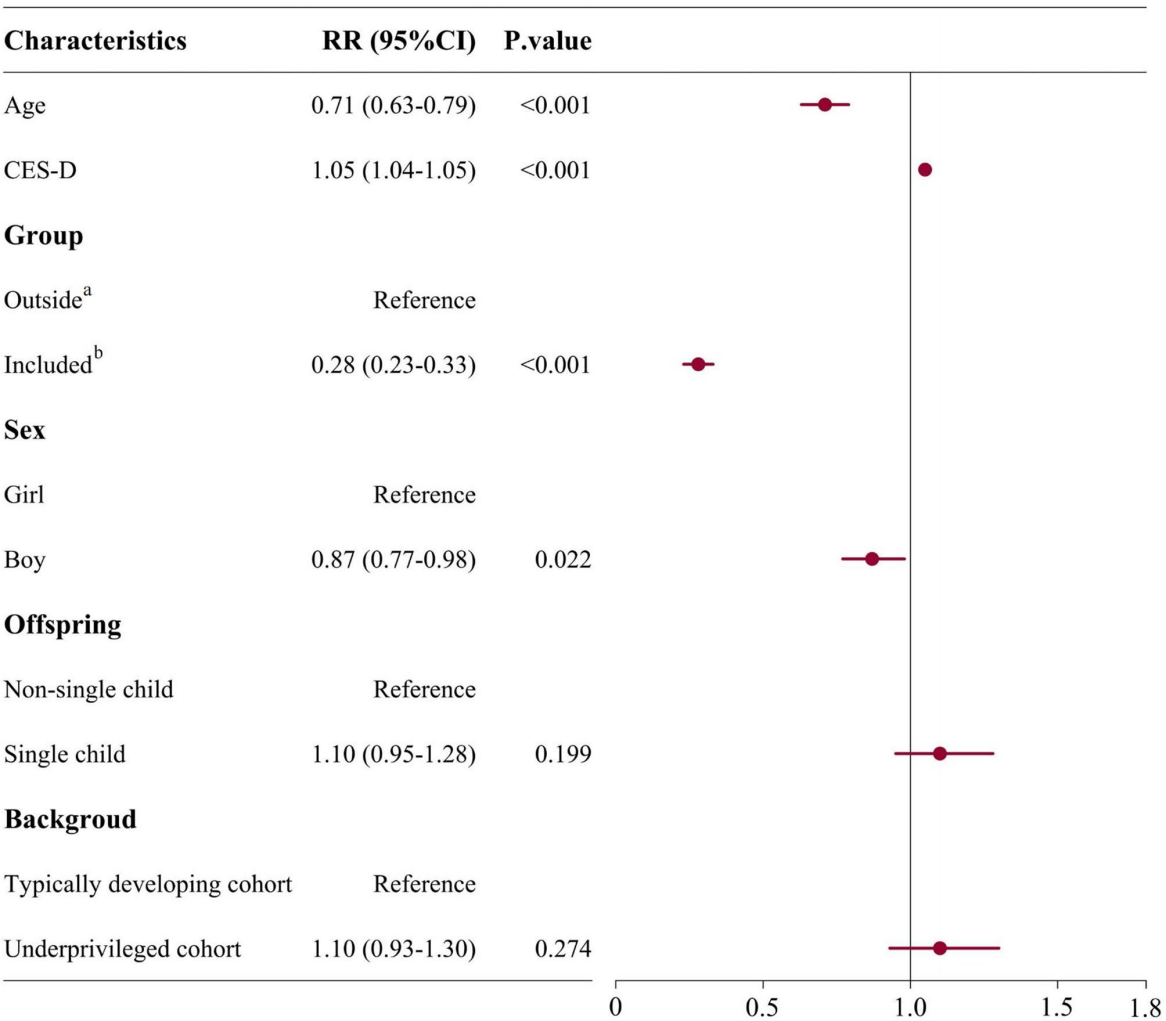
The adjusted relative risk (aRR) for the self-reporting suicide ideation at 1-year follow-up. **a***Outside* refers to children/adolescents not included in primary psychological healthcare system. **b***Included* refers to children/adolescents included in primary psychological healthcare system. The RRs were calculated by generalized linear mixed model. 95%CI = 95% Confidence Interval. CES-D = Center for Epidemiological Survey, Depression scale. When RR < 1, the variable is considered as protective factor for preventing suicide ideation. When RR > 1, the variable is considered as risk factor

As shown in Fig. 2, at the 0.5-year follow-up, after implementing the primary psychological healthcare system, 5.7% (95% CI: 5.3–6.1, 772/13,527) children/adolescents who were included in the system reported suicide ideation, whereas a higher incidence of suicide ideation was found for children/adolescents outside the system (8.1%, 95% CI: 7.4–8.9, 475/5,613). Results of GLMM showed significant effects (adjusted RR = 0.28, 95% CI 0.23–0.33; *p* < 0.001) of practicing this system on preventing suicide ideation among individuals included in the system, compared to those outside the system. Further, we observed the relative risk reduction (RRR) of 29.6% for individuals included in the system, compared to those outside the system (*p* < 0.01, Permutation test at *n* = 5,000; e.g., of 1000 children/adolescents, 57 reported suicide ideation in the included individuals vs. 81 in the excluded individuals). At the 1-year follow-up (Fig. 3), the incidence rate of reporting suicide ideation further decreased to 5.6% (95% CI: 5.3–6.0) for children/adolescents included in this system, but increased to 9.1% (95% CI 8.4–9.9) for those outside the system. The statistically significant 1-year-lasting effect (aRR = 0.28, 95% CI 0.23–0.33; *p* < 0.001, the same as 0.5-year follow-up) of implementing this system on preventing suicide ideation was still found. The RRR was observed to be increasing to 38.5% (*p* < 0.01, Permutation test at *n* = 5,000). In the sensitivity analysis, we also observed significantly lower risks of reporting suicidal ideation after practicing this primary psychological healthcare, both at the 0.5-year and 1-year follow-ups across all Imputed datasets (Table S7), thereby indicating the robustness of the above analysis.

In the subgroup analysis, at 0.5-year follow-up (May. 21, 2023), we found the statistically significant lower risks of reporting suicide ideation after practicing this primary psychological healthcare, in not only typically developing children/adolescents (adjusted RR 0.39, 95%CI 0.24–0.65), but also the underprivileged cohorts including CEDC (adjusted RR 0.28, 95%CI 0.21–0.37), “single-parent” children/adolescents (adjusted RR 0.24, 95%CI 0.16–0.38), “left-behind” children/adolescents (adjusted RR 0.26, 95%CI 0.21–0.33) and de facto unattended children/adolescents (adjusted RR 0.13, 95%CI 0.04–0.45), compared to children/adolescents under the same underprivileged condition but outside the system (Table 2; all *p* ≤ 0.001, Benjamini-Hochberg corrections). At 1-year follow-up (Oct. 29, 2023), such benefits were observed in typically developing cohort (adjusted RR 0.35, 95%CI 0.21–0.58), CEDC (adjusted RR 0.31, 95%CI 0.23–0.41), “single-parent” children/adolescents (adjusted RR 0.28, 95%CI 0.18–0.44) and “left-behind” children/adolescents (adjusted RR 0.25, 95%CI 0.20–0.31) only (all *p* < 0.001, Benjamini-Hochberg corrections; Table 2).

Despite such prominent and (1-year) long-term benefits, by using non-inferiority tests, we further examined whether this system unexpectedly incurs inequality of such primary health benefits in underprivileged children/adolescents, including the CEDC, “left-behind” and “single-parent” children/adolescents group, respectively, given their sample sizes reached statistical prerequisite. Results showed that, at a boundary value of 30% for the 0.5-year follow-up and 35% for the 1-year follow-up, the system’s effects were statistically non-inferior to those of CEDC, compared to typically developing individuals (all p_corrected_ ≤ 0.01, Table S5-9). Similarly, for “left-behind” children/adolescents, the effects were non-inferior at a boundary value of 30% for both the 0.5-year and 1-year follow-ups, when compared to typically developing individuals (all p_corrected_ ≤ 0.01, Table S5-9). For “single-parent” children/adolescents, the effects were inferior for 0.5-year follow-up and non-inferior at a boundary value of 45% for 1-year follow-up (p_corrected_ ≤ 0.05, Table S5-9), when compared to typically developing individuals. Exploratory analyses for the non-inferiority to other underprivileged cohorts can be found at Table S8-12.

The costs of implementing the primary psychological healthcare system were calculated separately by accounting groups (Supplemental results 5). The cost-effectiveness analysis indicated that the average expense for implementing this system amounted to ¥11.7 (approximately $1.6) per child/adolescent. The total expenditure of the healthcare, such as payments for healthcare staff, was $0.39 million. The expenditure for supportive services, such as APP maintenance, was $0.15 million.

## Discussion

This study provides the real-world evidence to illustrate the practical effects of establishing the primary psychological healthcare system on reducing the risks of children/adolescents’ suicide ideation in a lower- and middle-income city. By implementing such a “2 + 2 pattern” of the primary psychological healthcare system, the reported reduction of relative risk was nearly 30% compared to the individuals outside system at the 0.5-year follow-up and nearly 40% at the 1-year follow-up. In terms of the risks of psychological health inequality from this system, we found the relative risk reductions in not only the typically developing cohort but also the underprivileged subgroups, including CEDC, “left-behind” children/adolescents, and “single-parent” children/adolescents, whereas it failed to equally benefit orphan and unattended children/adolescents in our exploratory analysis. Together, these results substantiate the real-world and long-term (1-year) benefits of establishing this citywide, low-cost, and population-based “2 + 2 pattern” primary psychological healthcare system in reducing risks of suicide ideation among children/adolescents living in lower-middle-income areas, even under several specific underprivileged conditions.

The current study first examined the real-world effects of establishing a primary psychological healthcare system in controlling the risks of children/adolescents’ suicide ideation in a lower-middle-income city, and found nearly 40% relative risk reduction after implementing this system at a 1-year follow-up with a consistent adjusted RR. Although integrating primary healthcare and psychological services is considered one of the most viable options for preventing and solving children and adolescents’ suicide issues [23], the role of such a healthcare system in preventing suicide ideation among children/adolescents is still unclear. Supporting evidence drawn from a global meta-analysis reported a significant decrease in suicide ideation after integrating psychological healthcare into the primary system, which employed a post-primary school-based suicide prevention (PSSP) pattern, including awareness programs, screening, and interventions for high-risk children/adolescents [28]. However, contradictory results also illustrated the null effects of integrative psychological services on controlling the incidence of suicide attempts or severe suicide ideation at follow-ups (e.g., The Screening by Professionals program) [29], leading to concerns that such integrative primary psychological services may not be well enough to tackle “suicide epidemics”, especially for global children/adolescents. Therefore, the overarching and major contribution of the current study was to provide real-world evidence supporting the practical effects of establishing the pattern-specific primary psychological healthcare system in reducing the risks of children/adolescents’ suicide ideation.

The practical effects of implementing the primary psychological healthcare system in the current study could be attributed to its rectification of the following deficiencies. On the one hand, because of limited mental health resources (licensed psychiatrists, psychiatric registrars, and psychiatric nurses) and millions of community-dwelling individuals in need, the accessibility of psychological healthcare and medical services is still insufficient [30], despite the excessive mental health expenditures, especially in LMICs [15]. Therefore, the “2 + 2” psychological practice combined with large-scale, multi-center, and population-based design in the primary psychological healthcare system could directly ameliorate the universal accessibility of early identification of suicide ideation and early psychological services, covering all children/adolescents in need [31]. On the other hand, stigma associated with suicide-related issues among children and adolescents who are suffering from suicidal ideation handicaps them from seeking psychological support, leading to more serious outcomes (suicide attempt and suicide) [32]. These children/adolescents who are arrested by stigma usually lack mental health education and literacy as well as social support [33]. Hence, despite that the primary psychological healthcare system is categorized as low-cost project with “low-quality” concern, such brief psychological healthcare in this system could give them social support, enhance the mental health literacy about suicide related issues of children/adolescents, and help them to overcome the suicide crisis [34]. Additionally, we still need to evaluate the safety of the screening for suicide ideation among children/adolescents. A meta-analysis presented the prevalence of suicide ideation among children/adolescents in the western region of China as 14.1% (95% CI: 11.7–16.9) [35], which is higher than the rates observed both within and outside the healthcare system in the current study, indicating that such primary psychological healthcare systems do not induce or increase the risk of suicide ideation.

Another finding worthy to discuss is that we observed a potential moderate “inequality” for condition-specific underprivileged cohorts from this system, especially in unattended and orphan children/adolescents. Although enormous studies have indicated the negative relationship between disadvantageous circumstances and mental health of children/adolescents, we might deepen such relationship by illustrating that the adverse effects brought by these disadvantages were significantly deteriorated in unattended and orphan children/adolescents. The finding was consistent with previous study which indicated that the relationship between multidimensional poverty and mental health of children/adolescents was not always linear, but appeared to be steeper among individuals with extreme poverty [36]. On the one hand, such deterioration may be driven by more mental health challenges observed among unattended and orphan children/adolescents, such as prominently lower socioeconomic positions, peer bullying and relevant disadvantageous conditions (e.g., poverty, less caregiving and lacking access to education), which lead to higher risks for mental health problems than those of their peers [37]. Among these risk factors, shame is identified as one of the strongest potential risk factors for the mental health of children/adolescents [38]. Compared to other groups, orphan and unattended individuals experience a stronger and more persistent sense of self-stigma due to family structure and societal perceptions of differentness, which may impede them from help-seeking. On the other hand, barriers in healthcare system also significantly shape the mental health outcomes of these individuals. Previous practices linking to primary healthcare services for children/adolescents, are highly dependent on full-fledged social systems (e.g., family, school, community), but are hard to individually reach unattended ones outside these systems [39]. Additionally, relying solely on primary psychological healthcare system is insufficient to comprehensively address the various risk factors affecting the mental health of these individuals, such as economic hardships and educational barriers, and other dimensions of poverty existed in the underprivileged children/adolescents [36, 40].

Given the suboptimal benefits of this system for these unattended ones, we may prompt an urgent call for specific psychological healthcare towards these underprivileged children/adolescents without well-established parenting cares. More importantly, generalizing this system elsewhere may need to design specific solicitude for these underrepresented cohorts [41]. On the one hand, peer support programs could be implemented to provide emotional and psychological support for orphaned and unattended children. Evidence from previous study suggests that establishing school-wide peer interaction groups can effectively reduce social withdrawal and foster self-esteem among orphans, thereby enhancing their coping abilities [42]. Moreover, when individuals face emotional distress or mental health crises, peers can serve as a primary support system, offering timely assistance that may prevent suicidal behaviors [43]. On the other hand, through collaboration with community organizations or local charitable institutions, the primary psychological healthcare system can fully integrate and utilize the existing support networks and resources of these organizations, which can not only provide much-needed psychological support for orphans and unattended children/adolescents but also effectively address multidimensional poverty through the combination of financial aid, educational opportunities, and basic healthcare services [44, 45]. For instance, by cooperating with local educational and child welfare systems to provide educational subsidies or scholarships, the risk of school dropouts caused by economic difficulties among orphans and unattended children can be significantly reduced, thereby creating more equitable educational opportunities for them and promoting their psychological well-being [46].

### Implications and limitations

This study holds significant implications for the existing mental health policy framework. Although current mental health guidelines, such as the Mental Health Gap Action Programme (mhGAP), clearly indicate the crucial role of school-based psychological interventions for promoting the mental health of children/adolescents and preventing suicide, specific action plans and cost details are still lacking [47, 48]. Therefore, the CPHG provides a practical action plan for these guidelines, which centers on a school-based approach that encompasses the establishment of psychological monitoring mechanisms and the provision of primary psychological services, ensuring equitable access to psychological healthcare for all children/adolescents, particularly underprivileged groups. Furthermore, this “2 + 2” model of primary psychological healthcare system is instrumental in facilitating early interventions for “high-risk” children/adolescents to prevent the occurrence of suicidal behaviors. Additionally, research indicates that such early intervention model can effectively reduce economic costs and alleviate public health and economic burdens [49–51]. Thus, this low-cost “2 + 2” primary mental health care system provides a viable policy framework for managing the issue of child/adolescent suicide in low- and middle-income countries that are facing heavy public health burdens. Finally, the COVID-19 pandemic, a period marked by significant stress and uncertainty, has led to a widespread increase in medical visits among children and adolescents due to suicide-related symptoms and behaviors (e.g., suicide ideation, suicide attempts) [52–54]. This highlights the importance of targeted interventions and support systems [55], which align with the effectiveness of the primary psychological healthcare system we established in our study. Given the system’s success in such a high-stress environment, it may also be beneficial in a more stable, general societal context.

Despite its merits, several limitations in the current study should be considered. Firstly, these results were derived from observational cohorts in the policy changes to establish primary psychological healthcare (i.e., CPHG), but not yet from a standard Randomized Controlled Trial (RCT). Secondly, the protocol was registered only with IRB, which may potentially impact the transparency of the study. Thirdly, given the cost-saving design for this universal primary healthcare system, we indeed lacked clear evidence to validate the healthcare quality (e.g., psychological cares, psychological screenings, individual evaluations of this “2 + 2 pattern” practices). Fourthly, we only utilized depression symptoms as the risk factor in the initial psychological screening, due to the objective of achieving a high-sensitivity-but-balanced-cost primary psychological healthcare system. Fifthly, these data in the current study were drawn from a citywide, real-world population during the COVID-19 pandemic in China, leading to unpredictable losses in follow-ups, which might bias the observational results. Sixthly, this study reports outcomes based on follow-up intervals of 0.5 years and 1 year, with suicide ideation as the primary outcome. Future research could consider longer follow-up periods and additional secondary outcomes to comprehensively evaluate the CPHG’s sustained effects and holistic benefits. Seventhly, participants with severe suicidal ideation were excluded due to ethical considerations, which may have introduced selection bias, potentially underestimating the true burden. Future studies should address this limitation by including this critical group. Lastly, this study was conducted in a single city, Nanchong. Conducting similar studies in regions with diverse socioeconomic and cultural contexts could further enhance the generalizability of these findings. Nonetheless, the results provide critical insights and practical implications for regions with similar socioeconomic and cultural conditions.

## Conclusions

In conclusion, the current study provided empirical real-world evidence of the benefits of the population-based and low-cost primary psychological healthcare system in reducing citywide suicide ideation risks among children/adolescents in lower-middle-income areas. What’s more, we found nearly 30% relative risk reduction after establishing this system at 0.5-year follow-up, and even observed 1-year long-term benefits by showing nearly 40% at 1-year follow-up. On the other hand, the current study highlighted the risks of health inequality for the benefits of this system on underrepresented children/adolescents, especially for ones in underprivileged parenting care. Taken together, by conducting this observational, population-based and 1-year longitudinal study, we provided real-world evidence to substantiate the benefits of implementing the primary psychological healthcare system with “2 + 2 pattern” in reducing risks of children and adolescents’ suicide ideation, particularly in such lower-middle-income areas encompassing underrepresented ones who were in underprivileged conditions.

## Electronic supplementary material

Below is the link to the electronic supplementary material.

Supplementary Material 1

Supplementary Material 2

Supplementary Material 3

## Data Availability

All the follow-up data had been deposited into the Science Data Bank (ScienceDB, 10.57760/sciencedb.12150) for full accesses once being approved by the Data Regulation Office (CPHG-DRO). Form for applying the case-by-case approval could be found at the FigShare (10.6084/m9.figshare.24297532.v1). All the analyses were implemented by the commercial software (i.e., SPSS, IBM.Inc.) and open-source R packages (e.g., MICE). All code used in R software had been deposited into the Open Science Framework (OSF, https://osf.io/e5h3a/) for full accesses.

## Abbreviations

CEDC: Children/adolescents in especially difficult circumstance
CES-D: Center for Epidemiological Studies-Depression Scale
CPHG: Psychological Health Guard for Children and Adolescents Project of China
LMICs: Low- and middle-income countries
GLMM: Generalized linear mixed-effect model
RR: Relative risk
RRR: Relative risk reduction
