# Supplementary material for: The real-world evidence to the effects of primary psychological healthcare system in diluting risks of suicide ideation in underrepresented children/adolescents: an observational, multi-center, population-based, and longitudinal study
Source: Child Adolesc Psychiatry Ment Health. 2025 May 16;19:56. doi: 10.1186/s13034-025-00914-4 (PMC12085056; doi:10.1186/s13034-025-00914-4)
Supplement: Supplementary file 1 — Supplementary Material 1 [file 13034_2025_914_MOESM1_ESM.docx]

**Supporting information for**

**Title: The real-world evidence to the effects of primary psychological healthcare system in diluting risks of suicide ideation in underrepresented children/adolescents: an observational, multi-center, population-based, and longitudinal study**

**Running title:** The effect of primary psychological healthcare system in diluting risks of suicide ideation

**Authors:** Wei Li^1¶^, Xuerong Liu^1¶^, Qianyu Zhang^1,2¶^, Xiaobing Tian^3,4^, Xianyong An^4^, Jidong Ren^4^, Xiaodi Han^1^, Jingyu Lei^1^, Chang Shen^1^, Yanyan Li^1^, Ji Chen^5*^, Lei Xia^1^, Jingxuan Zhang^1^, Yi Wu^1^, Jie Gong^4^, Hai Lan^6^, Yan Wu^7^, Zhengzhi Feng^1*^, Zhiyi Chen^1,8*^

**Affiliation:**

^1^ Experimental Research Center of Medical and Psychological Science (ERC-MPS), School of Psychology, Third Military Medical University, Chongqing, China 400038

^2^ Department of Public Management, Chongqing University, Chongqing, China 400044

^3^ Department of Epidemiology and Public Health Statistics, North Sichuan Medical College, Nanchong, Sichuan, China 637000

^4^ Nanchong Psychosomatic Hospital (The Sixth People's Hospital of Nanchong), Nanchong, Sichuan, China 637000

^5^ Institute of Psychology and Behavioral Science, Shanghai Jiao Tong University, Shanghai, China 200030

^6^ Department of psychology, Sichuan Normal University, Chengdu, Sichuan, China 610068

^7^ School of Architecture , Zhengzhou University, Zhengzhou, Henan, China 450001

^8^ Key Laboratory of Cognition and Personality, Ministry of Education, Faculty of Psychology, Southwest University, Chongqing, China 400715

* Corresponding at: Zhiyi Chen (chenzhiyi@tmmu.edu.cn; TEL: +86 0 68771767) or Zhengzhi Feng (fzz@tmmu.edu.cn; TEL: +86 0 68771480), Experimental Research Center for Medical and Psychological Science, School of Psychology, Third Military Medical University; Gao Tan-Yan Main Street, Shapingba, Chongqing, P.R. China. Ji Chen (ji.chen@zju.edu.cn), Institute of Psychology and Behavioral Science, Shanghai Jiao Tong University, Shanghai, P.R. China.

¶ These authors contributed equally to this work

**CONTENT**

**Supplemental Methods ………………………………………………………………………………...4**

1. **Criteria of legally identified underprivileged conditions ……………...………………………. 4**
2. **Measures employed in the current study …………………………………………………………5**
3. **Psychological health-care center (s) ……………………………………………………………...7**
4. **The project details of CPHG system……………………………………………………………...8**
5. **Statistics …………………………………………………………………………………………..11**

**Supplemental Results …………………………………………………………………………..……..13**

1. **Overall incidence rates and crude Risk Ratio (RR) ……………………………………….…..13**
2. **Results of differential analyses between included and excluded participants…………………15**
3. **Results of sensitivity analysis……………………………………………………………………17**
4. **Results of non-inferiority tests …………………………………………………............………..18**
5. **Public budget statement……………………………….……………………………...23**
6. **STROBE statement …………………………………………………………………………..…..25**

**SUPPLEMENTAL METHODS**

**1.** **Criteria of legally identified underprivileged conditions**

Five underprivileged groups are identified based on the criteria established by Ministry of Civil Affairs of the People’s Republic of China, including adolescents in especially difficult circumstance (AEDC), “left-behind” adolescents, “single-parent” adolescents, de facto unattended adolescents, and orphans, which need legally mandated social care. The underprivileged ones have been categorized into one underprivileged group if he/she met the criteria mentioned above. The missing data are dummy coded. The criteria are presented as follows:

| De facto unattended children/adolescents[1] |
| --- |
| De facto unattended adolescents refer to individuals whose both parents meet one of the following conditions: severe disability, serious illness, serving a sentence in custody, mandatory isolation for drug rehabilitation, subject to other measures that restrict personal freedom, missing, revocation of guardianship qualification, deportation (expulsion) from the country; or in case one parent has passed away or is missing, the other parent meets one of the aforementioned conditions. |
| Orphan[2] |
| Orphan refers to an underage person under the age of 18 who has lost their parents or whose biological parents are untraceable, and this determination is made by the local civil affairs department at or above the county level based on relevant regulations and criteria. |
| Children/adolescents in especially difficult circumstance (CEDC) [3] |
| Adolescents in especially difficult circumstance refer to those who living in family containing a main member suffering from old age, physical weakness, living alone without care, or loss of working capacity; or adolescents who living in family with less than $8206 income per year (gathered from all family members.) Those adolescents are entitled “Wubao”, requiring a legally mandated social care including essentially material support, medical care, housing security, mental care, and funeral expenditure. |
| “Left-behind” children/adolescents[4] |
| “Left-behind” adolescents are left in the home without parenting care because parents (or either one) working away from home; or adolescent who lacked parents’ care in the home because they work too long to be unable for guardianship. |
| “Single-parent” children/adolescents[5] |
| “Single-parent” adolescents refer to those whose parents have separated or divorced. Others will have suffered the death of a parent. Some may well have only ever lived with one parent |

**2.** **Measures employed in the current study**

**Center for Epidemiological Studies-Depression Scale (CES-D)**

The CES-D is a self-report scale widely employed to screen depressive experiences in the large-scale epidemiological investigation, with well reliability and validity in the Chinese population[6]. The scale is coded by four-point style to describe the intensity of experiencing depressive feelings within 1 week, in which “0” is for “no experience”, “1” is for “rare”, “2” is for “frequent” and “3” is for “almost all the time”. The primary statistical indicator of the CES-D is the total score, which is the sum of the scores from 20 individual items. A total score of ≤15 indicates no symptoms of depression. A score of 16-19 suggests possible symptoms of depression. A score of ≥20 confirms the presence of depression symptoms.

The descriptions have been rephrased to keep consistent with other items in order to strengthen readability and understandings for adolescents **(Tab. S1)**.

**Self-reported item for single signaling suicide ideation**

The single item is scaled by five-point style to identify the single signaling suicide ideation within 2 weeks, with “0” for “no experience”, “1” for “rare”, “2” for “sometimes”, 3 for “often” and “4” is for “always”. Adolescents would be identified as having signaling severe suicide ideation if they answered: “often or always” (**Tab. S1**).

| **CES-D** |
| --- |
| My appetite was poor |
| I could not shark of the blues despite much supports from family and friends |
| I had trouble keeping my mind on what I was doing |
| I felt depressed |
| My sleep was restless |
| I felt sad |
| I could not get going |
| Nothing made me happy |
| I felt like a bad person than other ones |
| I lost interests in my life |
| I talk much less than usual |
| I had ever cried |
| I felt fear |
| I felt bad evaluations to me from other persons |
| I felt alone |
| I was tired to do anything all the time |
| I feel hopeless |
| I fail to go well for my life |
| I had a lot of trouble in many non-sense things |
| I felt no one like me |
| **Self-reported item for single signaling of suicide ideation** |
| have you ever felt hopeless for the future, giving rise to the idea of suicide? |

**Table S1. The items of scales employed in the current study.**

**3.** **Psychological health-care center (s)**

The children and adolescents recommend to transfer to clinical diagnosis and treatment have undergone such cares in government-sponsored mental health centers/hospitals. These centers/hospitals have been detailed as follows.

| **Regions** | **Centers (hospitals)** | **Class** | **Category** |
| --- | --- | --- | --- |
| Pengan | Nanchong City Physical and Mental Hospital | Grade III Level B hospital | public |
|  | Affiliated Hospital of North Sichuan Medical College | Grade III Level A hospital | public |
| Yilong | Yilong County People's Hospital | Grade III Level B hospital | public |
|  | Nanchong Central Hospital | Grade III Level A hospital | public |
| Langzhong | Langzhong People's Hospital | Grade III Level A hospital | public |
|  | Nanchong Central Hospital | Grade III Level A hospital | public |
| Gaoping | Nanchong City Physical and Mental Hospital | Grade III Level B hospital | public |
| Jialing | Nanchong City Physical and Mental Hospital | Grade III Level B hospital | public |
|  | Nanchong Second People's Hospital | Grade III Level A hospital | public |
| Nanbu | Nanbu County People's Hospital | Grade III Level A hospital | public |
| Yingshan | Nanchong City Physical and Mental Hospital | Grade III Level B hospital | public |
| Xichong | Affiliated Hospital of North Sichuan Medical College | Grade III Level A hospital | public |
|  | Nanchong Central Hospital | Grade III Level A hospital | public |

**Table S2. Details for center (hospital) to provide medical treatment/service for these children/adolescents**

**4. The project details of CPHG system**

The CPHG system adopted multiple procedures to maintain its implementation, including “2+2” psychological healthcare pattern, psychological healthcare training, psychological healthcare education and psychological healthcare management.

**Psychological healthcare training**

The psychological healthcare education aimed at enhancing the risk awareness of administrative leaders, the level of mental health cognitive awareness among primary and secondary school teachers, as well as the teaching abilities and intervention skills related to mental health education for mental health teachers, and promoting the deep integration of mental health education with daily teaching activities. The target individuals of psychological healthcare education included administrative leadership, head teacher, subject teacher, and full-time or part-time mental health teacher. The training methods included expert lectures, classroom demonstration teaching, hospital internships, and assistance with psychological intervention skills. Educational contents are as follows:

| Administrative leadership |
| --- |
| School Mental Health Education Workflow |
| Head teacher |
| Child and Adolescent Developmental Psychology, Positive Psychology, Interpersonal Communication, Emotion Management, Identification and Preliminary Handling of Common Psychological Issues among Students, Psychological Assistance Approaches, Parent-Child Relationships, and Family Mental Health Education. |
| Subject teacher |
| Child and Adolescent Developmental Psychology, Positive Psychology, Interpersonal Communication, and Emotion Management |
| Full-time or part-time mental health teacher |
| Child and Adolescent Developmental Psychology, Positive Psychology, Interpersonal Communication, Emotion Management, Identification and Preliminary Handling of Common Psychological Issues among Students, Psychological Assistance Approaches, Parent-Child Relationships, Family Mental Health Education, Introduction to School Mental Health Education, Psychological Assessment Techniques and Common Psychological Intervention Techniques, Construction of Campus Crisis Response Systems and Processes. |

**Psychological healthcare management**

Psychological healthcare management was conducted to supervise and manage the operation of the CPHG. As a supporting element, the psychological healthcare management mainly including establishment of mental health data platform, mental health records management, psychological crisis prediction and early warning, and data curation towards children/adolescents included in the CPHG system, as well as organization of psychological healthcare education, assessment and evaluation of healthcare staff.

**Psychological healthcare Education**

Through continuous, systematic, and professional popularization of mental health knowledge, promote the audience to fully understand the psychological characteristics and major mental health issues of minors at different stages, gradually master the basic skills of prevention and response. The target audience include parents, teachers, children/adolescents and volunteers working with minors.

Based on data collected during the previous work processes such as psychological health assessments of minors, teacher training, post-service for patients and children in distress, conduct statistical analysis. Combine specialized surveys, clinical work experience, and expert discussions to determine the cognitive abilities, cognitive levels, cognitive shortcomings, and main points of interest of the target audience, and establish the framework for the work content.

**Determine Communication Formats**

(1) Communication Platforms: TikTok, WeChat Official Accounts, WeChat Video Accounts

(2) Priority Order: Videos, comics, animations, popular science articles, and audio

(3) Frequency of Communication: 2 works per week

(4) Timing of Communication: Disseminate knowledge related to specific occasions such as new student enrollment, college entrance exams, major holidays, etc., in a targeted manner.

**5.** **Statistics**

**Generalized linear mixed model (GLMM)**

To explore the role of primary psychological healthcare system, we built generalized linear mixed model to minimize standard error[7].

$g\left( E\left( Y_{ij} | X_{i} \right) \right)= \beta_{0}+ X_{ij,1}x\beta_{1}+\ldots+ X_{ij,p}x\beta_{p}+ Z_{ij,1}xu_{1}+\ldots+ Z_{ij,q}xu_{q}$ (Equation 1)

Here, the dependent variable (DV) was estimated to the adjusted odd ratio (aRR) of exposing to CPHG. Age, sex, offspring, and CED-D have been modeled as confounders for adjustment. A random effect for clustering of adolescents within regions was accounted to capture variability between groups. These estimates have been done by “*stats*”, “*lmerTest*” and “*MASS*” packages of R (4.3.1) [8-10].

**Relative Risk Reduction (RRR)**

The purpose of calculating the relative risk reduction was to quantify the effectiveness of primary psychological healthcare system in preventing suicide ideation compared with the individuals unexposed to this system. The RRR was calculated by the following equation:

$RRR= \frac{incidence of suicide ideation \left( \mathrm{unexposed}^{1} \right)-incidence of suicide ideation \left( \mathrm{exposed}^{2} \right)}{incidence of suicide ideation \left( \mathrm{unexposed}^{1} \right)}\times100\%$ (Equation 2)

Notes: 1. *Unexposed* refers to the individuals unexposed to the CPHG system.

2. *Exposed* refers to the individuals exposed to the CPHG system.

**SUPPLEMENTAL RESULTS**

**1.** **Overall incidence rates and crude Risk Ratio (RR)**

|  |  | 0.5-year follow up | | |  | 1-year follow up | | |  |
| --- | --- | --- | --- | --- | --- | --- | --- | --- | --- |
|  |  | Case (%) | RR (95% CI) | P value |  | Case (%) | RR (95% CI) | P value |  |
| Typical developing cohort |  |  |  |  |  |  |  |  |  |
| Included^a^ |  | 129 (5.8%) | 0.89 (0.62-1.26) | 0.502 |  | 118 (5.3%) | 0.66 (0.47-0.92) | 0.015 |  |
| Outside^b^ |  | 44 (6.5%) | Reference |  |  | 53 (7.8%) | Reference |  |  |
| Adolescents in especially  difficult circumstance |  |  |  |  |  |  |  |  |  |
| Included^a^ |  | 288 (5.5%) | 0.66 (0.55-0.80) | <0.001 |  | 302 (5.7%) | 0.66 (0.54-0.79) | <0.001 |  |
| Outside^b^ |  | 179 (8.0%) | Reference |  |  | 189 (8.5%) | Reference |  |  |
| “Single-parent” adolescents |  |  |  |  |  |  |  |  |  |
| Included^a^ |  | 141 (7.3%) | 0.66 (0.50-0.88) | 0.004 |  | 115 (6.0%) | 0.52 (0.39-0.69) | <0.001 |  |
| Outside^b^ |  | 90 (10.6%) | Reference |  |  | 92 (10.8%) | Reference |  |  |
| “Left-behind” adolescents |  |  |  |  |  |  |  |  |  |
| Included^a^ |  | 534 (5.8%) | 0.73 (0.63-0.84) | <0.001 |  | 511 (5.6%) | 0.61 (0.53-0.70) | <0.001 |  |
| Outside^b^ |  | 296 (7.9%) | Reference |  |  | 334 (8.9%) | Reference |  |  |
| Unattended adolescents |  |  |  |  |  |  |  |  |  |
| Included^a^ |  | 15 (8.9%) | 0.44 (0.19-1.01) | 0.054 |  | 16 (9.5%) | 0.80 (0.31-2.04) | 0.637 |  |
| Outside^b^ |  | 11 (18.3%) | Reference |  |  | 7 (11.7%) | Reference |  |  |
| Orphan |  |  |  |  |  |  |  |  |  |
| Included^a^ |  | 7 (13.7%) | 0.42 (0.13-1.31) | 0.133 |  | 7 (13.7%) | 0.50 (0.16-1.60) | 0.244 |  |
| Outside^b^ |  | 8 (27.6%) | Reference |  |  | 7 (24.1%) | Reference |  |  |
| Total population before PS matching |  |  |  |  |  |  |  |  |  |
| Included^a^ |  | 772 (5.7%) | 0.68 (0.61-0.77) | <0.001 |  | 763 (5.6%) | 0.60 (0.53-0.67) | <0.001 |  |
| Outside^b^ |  | 457 (8.1%) | Reference |  |  | 511 (9.1%) | Reference |  |  |
| Total population after PS matching |  |  |  |  |  |  |  |  |  |
| Included^a^ |  | 157 (5.6%) | 0.82 (0.66-1.02) | 0.076 |  | 151 (5.4%) | 0.62 (0.50-0.77) | <0.001 |  |
| Outside^b^ |  | 189 (6.7%) | Reference |  |  | 236 (8.4%) | Reference |  |  |

**Table S3. Incidence of reporting suicide ideation in each cohort and crude RRs of CPHG to incidence of suicide ideation for each cohort in the 0.5-year and 1-year follow-ups.** CPHG = Psychological Health Guard for Children and Adolescents Project of China. PS matching = propensity score matching. a. *Included* refers to children/adolescents included in primary psychological healthcare system. b. *Outside* refers to children/adolescents not included in primary psychological healthcare system.

**2. Results of differential analyses between included and excluded participants**

In this section, we provide the basic demographics of the excluded individuals and examine the differences between excluded and included individuals, including age, sex, underprivileged background, and offspring. Considering the huge difference in sample size between the excluded samples and the included samples, even slight differences can lead to significant statistical differences. Therefore, we also used effect sizes to evaluate the magnitude of this difference (Cramer’s V for categorical variables, Cohen’s d for continuous variables). The values of Cramer’s V and Cohen’s d and their corresponding meanings are shown in Table S4-5. The results for the difference analysis are shown in Table S6.

| Cramer’ s V | Interpretation of Effect Size |
| --- | --- |
| 0 | No effect |
| 0.10 - 0.29 | Small effect |
| 0.30 - 0.49 | Medium effect |
| 0.50 - 0.69 | Large effect |
| 0.70 and above | Very large effect |

Table S4: Interpretation of Cramér’s V values and effect sizes.

| Cohen’s d | Interpretation of Effect Size |
| --- | --- |
| 0.2 ≤ d ≤ 0.5 | Small effect |
| 0.5 ≤ d ≤ 0.8 | Medium effect |
| d ≥ 0.8 | Large effect |

Table S5: Interpretation of Cohen’s d values and effect sizes.

|  | Excluded individuals (n=160866) | Included individuals (n=19140) | P value | Effect Size |
| --- | --- | --- | --- | --- |
| Age (M ± SD) | 14.40 ± 1.759 | 14.11 ± 1.507 | <0.001 | 0.167 |
| Sex |  |  |  |  |
| Boy | 84088 (52.27%) | 7362 (38.46%) | <0.001 | 0.085 |
| Girl | 76778 (47.73%) | 11778 (61.54%) |  |  |
| Offspring |  |  |  |  |
| Non-single child | 28799 (17.90%) | 3236 (16.91%) | <0.001 | 0.008 |
| Single child | 132077 (82.10%) | 15904 (83.09%) |  |  |
| Underprivileged background |  |  |  |  |
| Typically developing cohort | 33791 (21.01%) | 2901 (15.16%) | <0.001 | 0.080 |
| Underprivileged cohort | 126993 (78.94%) | 16074 (83.98%) |  |  |
| Missing | 82 (0.05 %) | 165 (0.86%) |  |  |

Table S6: Comparison of sociodemographic characteristics in included and excluded participants. Independent samples t test is utilized in the comparison of Age, while Chi-square Tests in other sociodemographic characteristics. The effect size for age is Cohen’s d, and the effect sizes for other variables are Cramer’ s V.

**3. Results of sensitivity analysis**

| **Datasets** | **N** | **0.5-year follow-up** | | |  | **1-year follow-up** | | |
| --- | --- | --- | --- | --- | --- | --- | --- | --- |
|  |  | **aRR^a^** | **95% CI** | **P value** |  | **aRR^a^** | **95% CI** | **P value** |
| **Dataset 1** |  |  |  |  |  |  |  |  |
| Included^b^ | 13,579 | 0.27 | 0.23-0.33 | <0.001 |  | 0.29 | 0.24-0.34 | <0.001 |
| Outside^c^ | 6,523 | Reference | | |  | Reference | | |
| **Dataset 2** |  |  |  |  |  |  |  |  |
| Included^b^ | 13,579 | 0.28 | 0.24-0.34 | <0.001 |  | 0.29 | 0.24-0.34 | <0.001 |
| Outside^c^ | 6,523 | Reference | | |  | Reference | | |
| **Dataset 3** |  |  |  |  |  |  |  |  |
| Included^b^ | 13,579 | 0.28 | 0.23-0.34 | <0.001 |  | 0.29 | 0.24-0.34 | <0.001 |
| Outside^c^ | 6,523 | Reference | | |  | Reference | | |
| **Dataset 4** |  |  | | |  |  | | |
| Included^b^ | 13,579 | 0.28 | 0.23-0.33 | <0.001 |  | 0.29 | 0.24-0.34 | <0.001 |
| Outside^c^ | 6,523 | Reference | | |  | Reference | | |
| **Dataset 5** |  |  | | |  |  | | |
| Included^b^ | 13,579 | 0.29 | 0.24-0.34 | <0.001 |  | 0.29 | 0.24-0.34 | <0.001 |
| Outside^c^ | 6,523 | Reference | | |  | Reference | | |
| **Original dataset** |  |  | | |  |  | | |
| Included^b^ | 13,527 | 0.28 | 0.23-0.33 | <0.001 |  | 0.28 | 0.23-0.33 | <0.001 |
| Outside^c^ | 5,613 | Reference | | |  | Reference | | |

Table S7: Sensitive analysis of the practical effects of implementing primary psychological healthcare system. Generalized linear mixed models were used for the analysis. a. Adjusted relative Risk, adjusted for all Sociodemographic characteristics (age, sex, offspring, and background) and depression estimated by Center for Epidemiological Survey, Depression scale (CES-D). b. Included refers to children/adolescents included in primary psychological healthcare system. c. Outside refers to children/adolescents not included in primary psychological healthcare system.

**4.** **Results of non-inferiority tests**

The non-inferiority tests were conducted to examine whether the primary psychological healthcare system provide equal benefits to all children/adolescents, especially those in underprivileged conditions. Given a little evidence to indicate the optimal non-inferior boundary, we tentatively limited this liberal boundary value from 30% to 50% of incidence rate of severe suicide ideation in typically developing individuals. We estimated the sample size to provide 90% statistical power for these non-inferiority tests, with the final significant level of 0.05 on two-side assumption. Results showed the effects of primary psychological healthcare system on CEDC were significantly non-inferior to developing individuals in the boundary value of 30% in T2 and 35% in T3 (all p_corrected_ ≤ 0.01). The effects on “left-behind” children/adolescents were significantly non-inferior to developing individuals in the boundary value of 30% in T2 and T3 (all p_corrected_ ≤ 0.01). These findings have been illustrated in the Table S8-12.

|  |  | 0.5-year follow-up (T2) | | | | |  | 1-year follow-up (T3) | | | | |  |
| --- | --- | --- | --- | --- | --- | --- | --- | --- | --- | --- | --- | --- | --- |
|  | N | Case (%) | Actual difference | Non-inferiority difference | Z | p |  | Case (%) | Actual difference | Non-inferiority difference | Z | p |  |
| Typically developing cohort | 2224 | 129 (5.8%) | - | - | - | - |  | 118 (5.3%) | - | - | - | - |  |
| CEDC | 5283 | 288 (5.5%) | -0.3% | 2.9% | -5.612 | <0.001 |  | 302 (5.7%) | 0.4% | 2.7% | -3.855 | <0.001 |  |
| “Left-behind” children/adolescents | 9160 | 534 (5.8%) | 0.0% | 2.9% | -5.185 | <0.001 |  | 511 (5.6%) | 0.3% | 2.7% | -4.401 | <0.001 |  |
| “Single-parent” children/adolescents | 1931 | 141 (7.3%) | 1.5% | 2.9% | -1.824 | 0.034 |  | 115 (6.0%) | 0.7% | 2.7% | -2.795 | 0.003 |  |
| De facto unattended child/adolescents | 168 | 15 (8.9%) | 3.1% | 2.9% | 0.425 | 0.664 |  | 16 (9.5%) | 4.2% | 2.7% | 0.852 | 0.803 |  |
| Orphan | 51 | 7 (13.7%) | 7.9% | 2.9% | 0.120 | 0.548 |  | 7 (13.7%) | 8.4% | 2.7% | 1.788 | 0.963 |  |

**Table S8. The results of non-inferiority tests for all the cohorts with a liberal boundary value of 50%.** All p values are not adjusted for Bonferroni correction. CEDC = Children/adolescents in especially difficult circumstance. The non-inferiority difference is 40% of incidence rate of typically developing cohort. It should be noted that CEDC , “left-behind” children/adolescents and “single-parent” children/adolescents are eligible for the expected samples of 1113 (T2) and 1225 (T3).

|  |  | 0.5-year follow-up (T2) | | | | |  | 1-year follow-up (T3) | | | | |  |
| --- | --- | --- | --- | --- | --- | --- | --- | --- | --- | --- | --- | --- | --- |
|  | N | Case (%) | Actual difference | Non-inferiority difference | Z | p |  | Case (%) | Actual difference | Non-inferiority difference | Z | p |  |
| Typically developing cohort | 2224 | 129 (5.8%) | - | - | - | - |  | 118 (5.3%) | - | - | - | - |  |
| CEDC | 5283 | 288 (5.5%) | -0.3% | 2.6% | -5.111 | <0.001 |  | 302 (5.7%) | 0.4% | 2.4% | -3.399 | <0.001 |  |
| “Left-behind” children/adolescents | 9160 | 534 (5.8%) | 0.0% | 2.6% | -4.661 | <0.001 |  | 511 (5.6%) | 0.3% | 2.4% | -3.911 | <0.001 |  |
| “Single-parent” children/adolescents | 1931 | 141 (7.3%) | 1.5% | 2.6% | -1.446 | 0.074 |  | 115 (6.0%) | 0.7% | 2.4% | -2.425 | 0.008 |  |
| De facto unattended child/adolescents | 168 | 15 (8.9%) | 3.1% | 2.6% | 0.272 | 0.607 |  | 16 (9.5%) | 4.2% | 2.4% | 0.996 | 0.840 |  |
| Orphan | 51 | 7 (13.7%) | 7.9% | 2.6% | 1.583 | 0.943 |  | 7 (13.7%) | 8.4% | 2.4% | 1.870 | 0.969 |  |

**Table S9. The results of non-inferiority tests for all the cohorts with a liberal boundary value of 45%.** All p values are not adjusted for Bonferroni correction. CEDC = Children/adolescents in especially difficult circumstance. The non-inferiority difference is 40% of incidence rate of typically developing cohort. It should be noted that CEDC , “left-behind” children/adolescents and “single-parent” children/adolescents are eligible for the expected samples of 1374 (T2) and 1512 (T3).

|  |  | 0.5-year follow-up (T2) | | | | |  | 1-year follow-up (T3) | | | | |  |
| --- | --- | --- | --- | --- | --- | --- | --- | --- | --- | --- | --- | --- | --- |
|  | N | Case (%) | Actual difference | Non-inferiority difference | Z | p |  | Case (%) | Actual difference | Non-inferiority difference | Z | p |  |
| Typically developing cohort | 2224 | 129 (5.8%) | - | - | - | - |  | 118 (5.3%) | - | - | - | - |  |
| CEDC | 5283 | 288 (5.5%) | -0.3% | 2.3% | -4.610 | <0.001 |  | 302 (5.7%) | 0.4% | 2.1% | -2.942 | 0.002 |  |
| “Left-behind” children/adolescents | 9160 | 534 (5.8%) | 0.0% | 2.3% | -4.138 | <0.001 |  | 511 (5.6%) | 0.3% | 2.1% | -3.420 | <0.001 |  |
| “Single-parent” children/adolescents | 1931 | 141 (7.3%) | 1.5% | 2.3% | -1.067 | 0.143 |  | 115 (6.0%) | 0.7% | 2.1% | -2.055 | 0.020 |  |
| De facto unattended child/adolescents | 168 | 15 (8.9%) | 3.1% | 2.3% | 0.425 | 0.664 |  | 16 (9.5%) | 4.2% | 2.1% | 1.140 | 0.873 |  |
| Orphan | 51 | 7 (13.7%) | 7.9% | 2.3% | 1.669 | 0.952 |  | 7 (13.7%) | 8.4% | 2.1% | 1.952 | 0.975 |  |

**Table S10. The results of non-inferiority tests for all the cohorts with a liberal boundary value of 40%.** All p values are not adjusted for Bonferroni correction. CEDC = Children/adolescents in especially difficult circumstance. The non-inferiority difference is 40% of incidence rate of typically developing cohort. It should be noted that CEDC , “left-behind” children/adolescents and “single-parent” children/adolescents are eligible for the expected samples of 1428 (T2) and 1678 (T3).

|  |  | 0.5-year follow-up (T2) | | | | |  | 1-year follow-up (T3) | | | | |  |
| --- | --- | --- | --- | --- | --- | --- | --- | --- | --- | --- | --- | --- | --- |
|  | N | Case (%) | Actual difference | Non-inferiority difference | Z | p |  | Case (%) | Actual difference | Non-inferiority difference | Z | p |  |
| Typically developing cohort | 2224 | 129 (5.8%) | - | - | - | - |  | 118 (5.3%) | - | - | - | - |  |
| CEDC | 5283 | 288 (5.5%) | -0.3% | 2.0% | -4.109 | <0.001 |  | 302 (5.7%) | 0.4% | 1.9% | -2.486 | 0.006 |  |
| “Left-behind” children/adolescents | 9160 | 534 (5.8%) | 0.0% | 2.0% | -3.614 | <0.001 |  | 511 (5.6%) | 0.3% | 1.9% | -2.929 | 0.002 |  |
| “Single-parent” children/adolescents | 1931 | 141 (7.3%) | 1.5% | 2.0% | -0.689 | 0.245 |  | 115 (6.0%) | 0.7% | 1.9% | -1.684 | 0.046 |  |
| De facto unattended child/adolescents | 168 | 15 (8.9%) | 3.1% | 2.0% | -0.577 | 0.718 |  | 16 (9.5%) | 4.2% | 1.9% | 1.284 | 0.900 |  |
| Orphan | 51 | 7 (13.7%) | 7.9% | 2.0% | 1.756 | 0.960 |  | 7 (13.7%) | 8.4% | 1.9% | 2.034 | 0.979 |  |

**Table S11. The results of non-inferiority tests for all the cohorts with a liberal boundary value of 35%.** All p values are not adjusted for Bonferroni correction. CEDC = Children/adolescents in especially difficult circumstance. The non-inferiority difference is 35% of incidence rate of typically developing cohort. It should be noted that CEDC and “left-behind” children/adolescents are eligible for the expected samples of 2320 (T2) and 2850 (T3).

|  |  | 0.5-year follow-up | | | | |  | 1-year follow-up | | | | |  |
| --- | --- | --- | --- | --- | --- | --- | --- | --- | --- | --- | --- | --- | --- |
|  | N | Case (%) | Actual difference | Non-inferiority difference | Z | p |  | Case (%) | Actual difference | Non-inferiority difference | Z | p |  |
| Typically developing cohort | 2224 | 129 (5.8%) | - | - | - | - |  | 118 (5.3%) | - | - | - | - |  |
| CEDC | 5283 | 288 (5.5%) | -0.3% | 1.7% | -3.608 | <0.001 |  | 302 (5.7%) | 0.4% | 1.6% | -2.030 | 0.021 |  |
| “Left-behind” children/adolescents | 9160 | 534 (5.8%) | 0.0% | 1.7% | -3.090 | 0.001 |  | 511 (5.6%) | 0.3% | 1.6% | -2.439 | 0.007 |  |
| “Single-parent” children/adolescents | 1931 | 141 (7.3%) | 1.5% | 1.7% | -0.311 | 0.378 |  | 115 (6.0%) | 0.7% | 1.6% | -1.314 | 0.094 |  |
| De facto unattended child/adolescents | 168 | 15 (8.9%) | 3.1% | 1.7% | 0.729 | 0.767 |  | 16 (9.5%) | 4.2% | 1.6% | 1.428 | 0.923 |  |
| Orphan | 51 | 7 (13.7%) | 7.9% | 1.7% | 1.842 | 0.967 |  | 7 (13.7%) | 8.4% | 1.6% | 2.116 | 0.983 |  |

**Table S12. The results of non-inferiority tests for all the cohorts with a liberal boundary value of 30%.** All p values are not adjusted for Bonferroni correction. CEDC = Children/adolescents in especially difficult circumstance. The non-inferiority difference is 30% of incidence rate of typically developing cohort. It should be noted that CEDC and “left-behind” children/adolescents are eligible for the expected samples of 5066 in T2, and only “left-behind” children/adolescents are eligible for the expected samples of 7219 in T3.

**5.** **Public budget statement**

As legal statement required, we limited to disclose the budget statement that permitted by accounting firms, to favor the understandings of the estimates for costs. Please see as follow:

| **Source of Funding** | |
| --- | --- |
| 1.Project Funding Application | 2 million |
| 2.Own Funds |  |
| 3.Other Funding Support | 1.5 million (Nanchong Civil Affairs) |
| **The purpose of this funding application** | |
| 1. Operating Expenses | 2 million（100%） |
| 1. Labor Costs | 700 thousand（35%） |
| A. Supervision Fee | 10,0000 RMB (5%) is primarily allocated for evaluation, training, post-assessment, inviting experts for project process consultation, and expert supervision expenses. |
| B. Social Worker Fee | 60,000 RMB (3%) is allocated to provide subsidies for social workers participating in the project. |
| C. Volunteer Allowance | 60,000 RMB（3%）is allocated to provide subsidies for the volunteers participating in the project. |
| D. Indirect Costs | 480,000 RMB（24%）is allocated for performance-based expenses of personnel involved in the project. |
| 1. Project Activity Expenditures | 1.3 million（65%） |
| A. Adolescent Psychological General Assessment | 360,000 RMB (18%) is primarily allocated for the bi-annual psychological assessment of children and adolescents in Nanchong City. |
| B. Training for Full-time and Part-time Psychology Teachers | 240,000 RMB (12%) is primarily allocated for the training and professional development of full-time and part-time mental health teachers in the entire city. |
| C. Post-assessment Services | 440,000 RMB (22%) is primarily allocated for psychological interviews, counseling referrals, and follow-up for disadvantaged children and post-assessment. |
| D. Popularization of Mental Health Knowledge | 140,000 RMB (7%) is primarily allocated for the writing of popular science scripts, expert fees, recording costs, and campus popular science activities. |
| E. Project Publicity | 120,000 RMB (6%) is primarily allocated for project promotion and publicity. |
| 1. Others |  |
| **The use of own and other project funds** | |
| The municipal matching fund is 1.5 million yuan. The assessment costs for minors in the entire city, excluding disadvantaged children (73.34%), as well as indirect expenses of 400,000 yuan (26.66%). | |

**Table S10. The cost of implementing primary psychological healthcare system.**

**6. STROBE statement**

STROBE Statement—Checklist of items that should be included in reports of ***cohort studies***

|  | Item No | Recommendation | Section and Paragraph numbers |
| --- | --- | --- | --- |
| **Title and abstract** | 1 | (*a*) Indicate the study’s design with a commonly used term in the title or the abstract | Title |
|  |  | (*b*) Provide in the abstract an informative and balanced summary of what was done and what was found | Abstract |
| Introduction | | |  |
| Background/rationale | 2 | Explain the scientific background and rationale for the investigation being reported | Introduction (paragraphs 1-3) |
| Objectives | 3 | State specific objectives, including any prespecified hypotheses | Introduction (paragraph 4) |
| Methods | | |  |
| Study design | 4 | Present key elements of study design early in the paper | Methods- Study design and participants (paragraphs 1-3) |
| Setting | 5 | Describe the setting, locations, and relevant dates, including periods of recruitment, exposure, follow-up, and data collection | Introduction (paragraph 4) and Methods-Study design and participants (paragraphs 1) |
| Participants | 6 | (*a*) Give the eligibility criteria, and the sources and methods of selection of participants. Describe methods of follow-up | Methods- Study design and participants (paragraphs 2-3) |
|  |  | (*b*) For matched studies, give matching criteria and number of exposed and unexposed | Methods- Statistical analysis (paragraphs 4) |
| Variables | 7 | Clearly define all outcomes, exposures, predictors, potential confounders, and effect modifiers. Give diagnostic criteria, if applicable | Methods- Study design and participants (paragraphs 2-3), and Outcome |
| Data sources/ measurement | 8* | For each variable of interest, give sources of data and details of methods of assessment (measurement). Describe comparability of assessment methods if there is more than one group | Methods-Procedure |
| Bias | 9 | Describe any efforts to address potential sources of bias | Methods- Statistical analysis (paragraph 2 and 4) |
| Study size | 10 | Explain how the study size was arrived at | Methods- Study design and participants (paragraph 2) |
| Quantitative variables | 11 | Explain how quantitative variables were handled in the analyses. If applicable, describe which groupings were chosen and why | Methods- Study design and participants (paragraph 3), and Statistical analysis (paragraphs 1-3) |
| Statistical methods | 12 | (*a*) Describe all statistical methods, including those used to control for confounding | Methods- Statistical analysis (paragraphs 1-4) |
|  |  | (*b*) Describe any methods used to examine subgroups and interactions | Methods- Statistical analysis (paragraphs 2-3) |
|  |  | (*c*) Explain how missing data were addressed | Methods- Statistical analysis (paragraph 2) |
|  |  | (*d*) If applicable, explain how loss to follow-up was addressed | N.A. |
|  |  | (*e*) Describe any sensitivity analyses | Methods- Statistical analysis (paragraph 4) |
| Results | | |  |
| Participants | 13* | (a) Report numbers of individuals at each stage of study—eg numbers potentially eligible, examined for eligibility, confirmed eligible, included in the study, completing follow-up, and analysed | Results (paragraph 1) and Fig 1 |
|  |  | (b) Give reasons for non-participation at each stage | Results (paragraph 1) and Fig 1 |
|  |  | (c) Consider use of a flow diagram | Fig 1 |
| Descriptive data | 14* | (a) Give characteristics of study participants (eg demographic, clinical, social) and information on exposures and potential confounders | Table 1 |
|  |  | (b) Indicate number of participants with missing data for each variable of interest | Table 1 |
|  |  | (c) Summarise follow-up time (eg, average and total amount) | Introduction (paragraph 4) |
| Outcome data | 15* | Report numbers of outcome events or summary measures over time | Results (paragraph 2) and Table 2 |
| Main results | 16 | (*a*) Give unadjusted estimates and, if applicable, confounder-adjusted estimates and their precision (eg, 95% confidence interval). Make clear which confounders were adjusted for and why they were included | Methods- Statistical analysis (paragraph 2) Results (paragraph 2) |
|  |  | (*b*) Report category boundaries when continuous variables were categorized | N.A. |
|  |  | (*c*) If relevant, consider translating estimates of relative risk into absolute risk for a meaningful time period | N.A. |
| Other analyses | 17 | Report other analyses done—eg analyses of subgroups and interactions, and sensitivity analyses | Results (paragraphs 3-5) |
| Discussion | | |  |
| Key results | 18 | Summarise key results with reference to study objectives | Discussion (paragraph 1) |
| Limitations | 19 | Discuss limitations of the study, taking into account sources of potential bias or imprecision. Discuss both direction and magnitude of any potential bias | Discussion (paragraph 5) |
| Interpretation | 20 | Give a cautious overall interpretation of results considering objectives, limitations, multiplicity of analyses, results from similar studies, and other relevant evidence | Discussion (paragraph 2-4) |
| Generalizability | 21 | Discuss the generalizability (external validity) of the study results | Discussion (paragraph 4) |
| Other information | | |  |
| Funding | 22 | Give the source of funding and the role of the funders for the present study and, if applicable, for the original study on which the present article is based | Methods- Role of the funding source |

*Give information separately for exposed and unexposed groups.

**References**

[1] Ministry of Civil Affairs of the People's Republic of China, Ministry of Public Securi-ty of the People's Republic of China, Ministry of Finance of the People's Republic of China. Notice on Further Improving the Relevant Work of de facto unattended children. 2020 December 24 [Cited 2024 January 10]. Available from: <https://www.gov.cn/zhengce/zhengceku/2021-01/26/content_5582578.htm>.

[2] General Office of the State Council of the People's Republic of China. State Coun-cil Office on Strengthening Orphan Protection Work Opinions. 2010 November 16 [Cited 2024 January 10]. Available from: <https://www.gov.cn/gongbao/content/2010/content_1754115.htm>.

[3] Ministry of Civil Affairs of the People's Republic of China. The order of Ministry of Civil Affairs of the People's Republic of China. 2010 October 22 [Cited 2024 January 10]. Available from: <https://www.gov.cn/gongbao/content/2011/content_1808597.htm>.

[4] State Council of the People's Republic of China. State Council's Opinion on Stren-gthening the Care and Protection of Left-behind Children in Rural Areas. 2016 February 14 [Cited 2024 January 10]. Available from: <https://www.gov.cn/zhengce/content/2016-02/14/content_5041066.htm>.

[5] Burghes L. What happens to the children of single parent families? Bmj (1994) 308(6937): 1114-5. DOI: 10.1136/bmj.308.6937.1114.

[6] Zhang J, Sun W, Kong Y, Wang C. Reliability and validity of the Center for Epidemiological Studies Depression Scale in 2 special adult samples from rural China. Compr Psychiatry (2012) 53(8): 1243-51. DOI: 10.1016/j.comppsych.2012.03.015.

[7] Yu Z, Guindani M, Grieco SF, Chen L, Holmes TC, Xu X. Beyond t test and ANOVA: applications of mixed-effects models for more rigorous statistical analysis in neuroscience research. Neuron (2022) 110(1): 21-35. DOI: 10.1016/j.neuron.2021.10.030.

[8] R Core Team. R: A language and environment for statistical computing. R Foundation for Statistical Computing, Vienna, Austria. 2022. https://www.r-project.org/

[9] Venables WN, Ripley BD. Modern Applied Statistics with S, Fourth edition. New York: Springer. 2002. https://www.stats.ox.ac.uk/pub/MASS4/.

[10] Kuznetsova A, Brockhoff PB, Christensen RHB. lmerTest Package: Tests in Linear Mixed Effects Models. Journal of Statistical Software, 2017; 82(13): 1 - 26. DOI: 10.18637/jss.v082.i13
