# Supplementary material for: The real-world evidence to the effects of primary psychological healthcare system in diluting risks of suicide ideation in underrepresented children/adolescents: an observational, multi-center, population-based, and longitudinal study
Source: Child Adolesc Psychiatry Ment Health. 2025 May 16;19:56. doi: 10.1186/s13034-025-00914-4 (PMC12085056; doi:10.1186/s13034-025-00914-4)
Supplement: Supplementary file 3 — Supplementary Material 3 [file 13034_2025_914_MOESM3_ESM.docx]

Project Work Manual

(Hospital Sector Work Framework)

To thoroughly implement General Secretary Xi Jinping's important instructions on the protection of minors, in early June 2022, in response to prominent issues such as the psychological health, myopia, dental caries, and scoliosis of minors in Sichuan Province, Sichuan Province proposed for the first time to carry out a pilot project for the "Bright Eyes and White Teeth, Righteous Heart and Upright Body" health project. It specifically outlined that Nanchong City would carry out a pilot project focusing on the psychological health of minors under the "Righteous Heart" health project. In accordance with the project requirements, the Nanchong City Minor Protection Center, the Nanchong City Civil Affairs Bureau, and the Nanchong City Mental Health Hospital jointly formulated the "Nanchong City 'Righteous Heart' Health Project Implementation Plan" (Nanwei Office [2022] No. 9). In accordance with the requirements of the plan, combining on-site investigations by the project team, expert discussions, and trial schemes in various work processes, this work manual was developed.

**1. Project Objective**

Establish the "575" service model for promoting the mental health of minors, comprehensively enhancing the mental health level of minors.

5-party linkage: Construct a mental health service model that connects "institutions + schools + families + communities + charitable forces" in five aspects.

7 major actions: Carry out "seven major actions" including site construction, health education, environment creation, health promotion, health care, capacity building, and service system improvement.

5 project goals: Implement institutionalization through multiple-party linkage, standardize work processes, digitize work throughout, make health decisions precise, and diversify fundraising channels, among other "five aspects."

**2. Project Team**

**2.1 Expert Team**

14 medical guidance experts are responsible for program approval, quality control, and academic research. They come from 8 universities or research institutions including West China School of Public Health at Sichuan University, Peking University, and the Institute of Psychology at the Chinese Academy of Sciences.

There are 19 members in the project guidance expert group (Nanchong City), responsible for program implementation, standard development, impact assessment, and teacher training. These experts come from institutions such as the National Health Commission's Institute of Science and Technology, China West Normal University, North Sichuan Medical College, Southwest Petroleum University, and public mental health institutions.

**2.2 Medical Technology Team**

Responsible for psychological health screening, post-examination medical services, and teacher training.

A total of 17 clinical psychologists and psychiatrists were selected from institutions such as Nanchong Mental Hospital, Nanchong Mental Health Center, and the affiliated hospital of North Sichuan Medical College. They are responsible for psychological health screening, post-examination medical services, and teacher training.

**2.3 On-site work team**

Engaged in community rehabilitation management, home visits, initial interviews, psychological counseling, and offline popular science activities. A total of 10 mental health social organizations are involved. With the Nanchong Mental Health Association as the core, following the principle of one institution per district, the "Evaluation Standards for the Capacity of Social Organizations to Participate in Mental Health Services for Minors" and the "Conditions for Social Organizations to Participate in Services for Minors in Nanchong" are used for pre-audit.

**3. On-site work team**

A total of 10 working groups are established, including: 1 project management group, 5 core working groups for building psychological health assessments, post-medical services, site construction, training, and mental health popularization; 2 coordinating groups for secretarial and logistical support, as well as 2 innovative support groups for publicity and academic support.


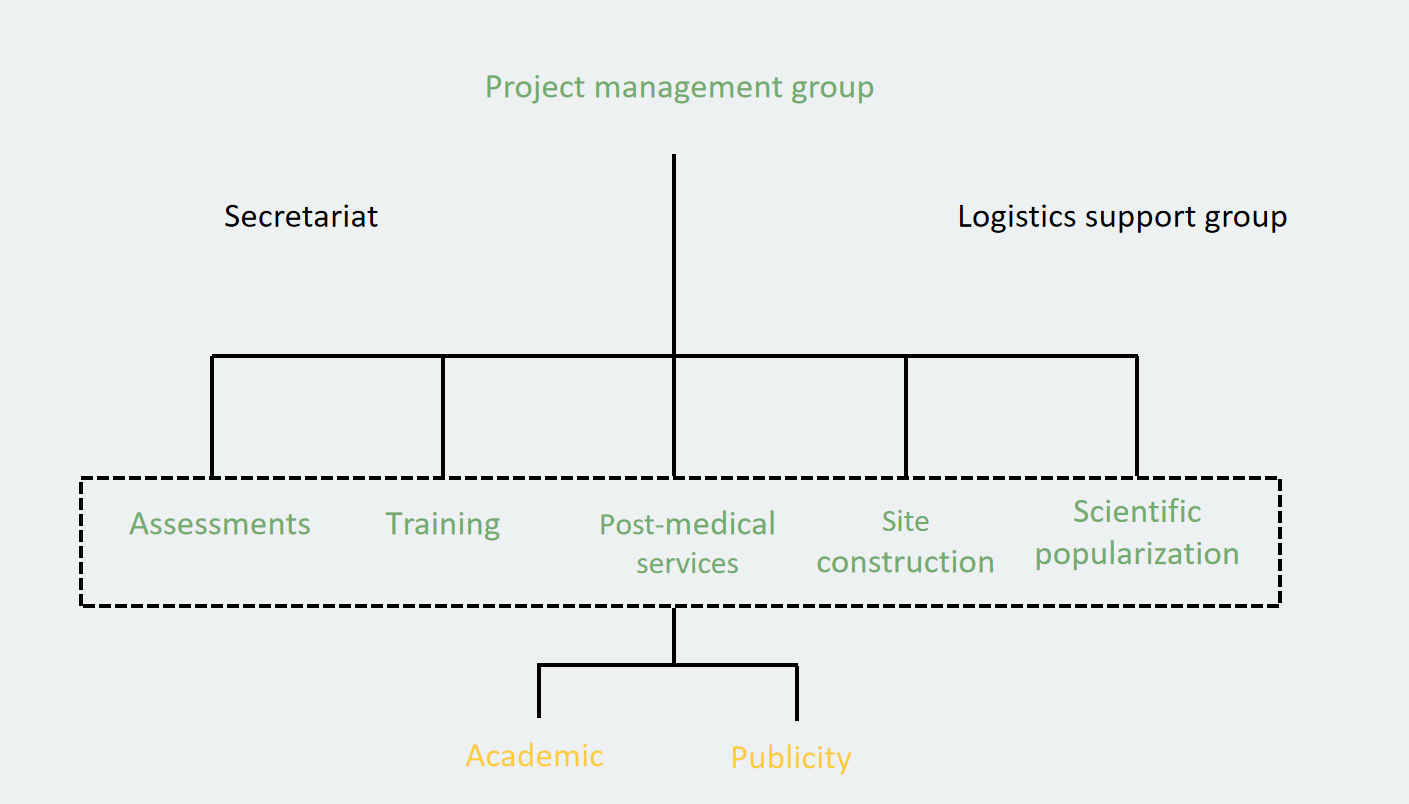


**4. Key Tasks**

**4.1 Mental Health Screening**

The “2+2” psychological healthcare pattern

**4.1.1 Project objectives**

Master the psychological health status of minors and identify individuals with psychological health problems, establish a data platform for children and adolescents' psychological health in Nanchong City, as well as a model for predicting and warning of psychological crises.

**4.1.2 Project Framework**

The first-round screening: Screening students with depressive tendencies;

The Second-round screening: In the population with depressive tendencies, further determine suicidal ideation through assessment results;

Structured initial interview: For students with severe suicidal ideation indicated by assessments or recommended by class teachers, a structured initial interview is conducted by school mental health teachers;

Discriminative interview: For students identified by initial interviews as needing further discrimination, parents or guardians are informed by mental health teachers or class teachers, and with their informed consent, a psychiatrist from a public psychiatric hospital is dispatched to the school for a second discriminative interview.


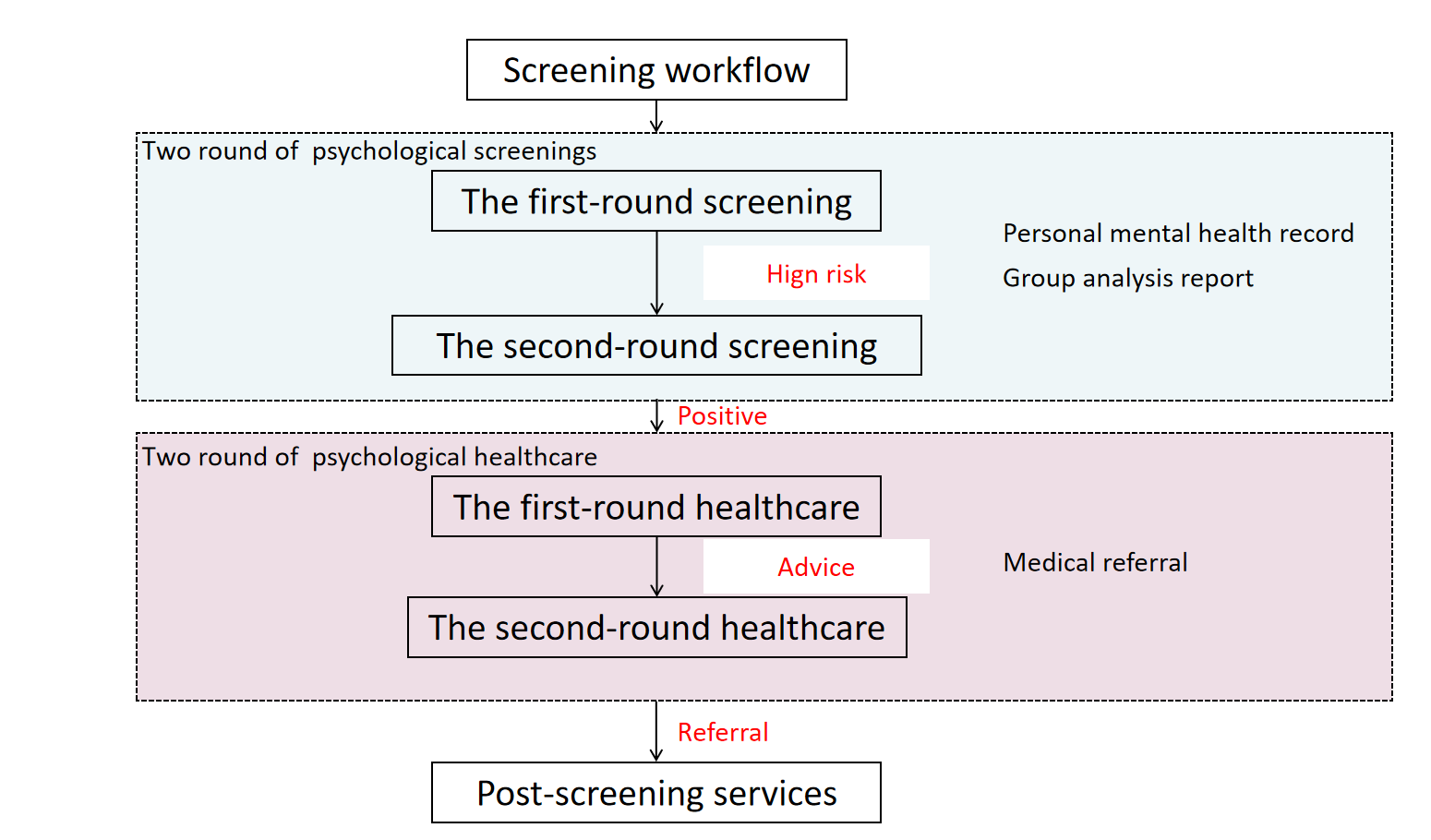


**4.1.3** The evaluation scales

| **Center for Epidemiological Studies-Depression Scale (CES-D)** |
| --- |
| My appetite was poor |
| I could not shark of the blues despite much supports from family and friends |
| I had trouble keeping my mind on what I was doing |
| I felt depressed |
| My sleep was restless |
| I felt sad |
| I could not get going |
| Nothing made me happy |
| I felt like a bad person than other ones |
| I lost interests in my life |
| I talk much less than usual |
| I had ever cried |
| I felt fear |
| I felt bad evaluations to me from other persons |
| I felt alone |
| I was tired to do anything all the time |
| I feel hopeless |
| I fail to go well for my life |
| I had a lot of trouble in many non-sense things |
| I felt no one like me |
| **Self-reported item for single signaling of suicide ideation** |
| have you ever felt hopeless for the future, giving rise to the idea of suicide? |

**4.1.4 Pre-test**

Select 2 middle schools and 2 high schools for pre-testing, analyze the reliability and validity, acceptability, and based on the pre-test results, establish the formal evaluation workflow.

**4.1.5 Assessment system**

The "Three-level Comprehensive Mental Health Service Platform for Minors" developed by the National Health Commission's Institute of Science and Technology covers important areas such as psychological health assessment, psychological health record management, psychological crisis prediction and early warning, supervision of high-risk individuals, referral treatment green channel, psychological health education platform, and collaborative data and information sharing. It supports different assessment modes both online and offline.

**4.1.6 Assessment Work Arrangement**

Classes are conducted in two rounds per year, with teachers and students participating simultaneously. Special education schools and welfare institutions organize separate activities.

First Semester: Starting from the third week after the beginning of the school year, lasting for 2 weeks;

Second Semester: Starting from the fourth week after the beginning of the school year, lasting for 3 weeks (including the National Day holiday).

**4.1.7 Information Reporting**

At three levels of school, county, and city, write analysis reports by analyzing data. At the same time, at the levels of junior high school and high school, establish predictive and early warning models for mental health issues among minors.

**4.2 Medical services**

End-to-end closed-loop management.

**4.2.1 Work Object**

After the differential interview by clinical physicians, a list of students who need to receive medical services is proposed. The list will be sent back to the mental health teacher, who will inform the parents. After obtaining the parents' informed consent, the children with illnesses will be referred to the hospital for outpatient or inpatient treatment.

**4.2.2 Work objectives**

Promote the recovery of the child through comprehensive services such as psychological intervention, medication and non-pharmacological therapy, and inpatient treatment.

**4.2.3 Workflow**

Based on the patient's condition, doctors make medical recommendations such as close observation, non-drug therapy, drug therapy, or hospitalization, and carry out related medical services.

After treatment, rehabilitated children are transferred to their homes or communities to continue receiving community rehabilitation management. Community mental health specialized services are provided by social psychological service organizations that have been qualified and confirmed by the project team. The organization designates a person to take over the management work, while also completing community registration, conducting home visits, psychological counseling, and other work.


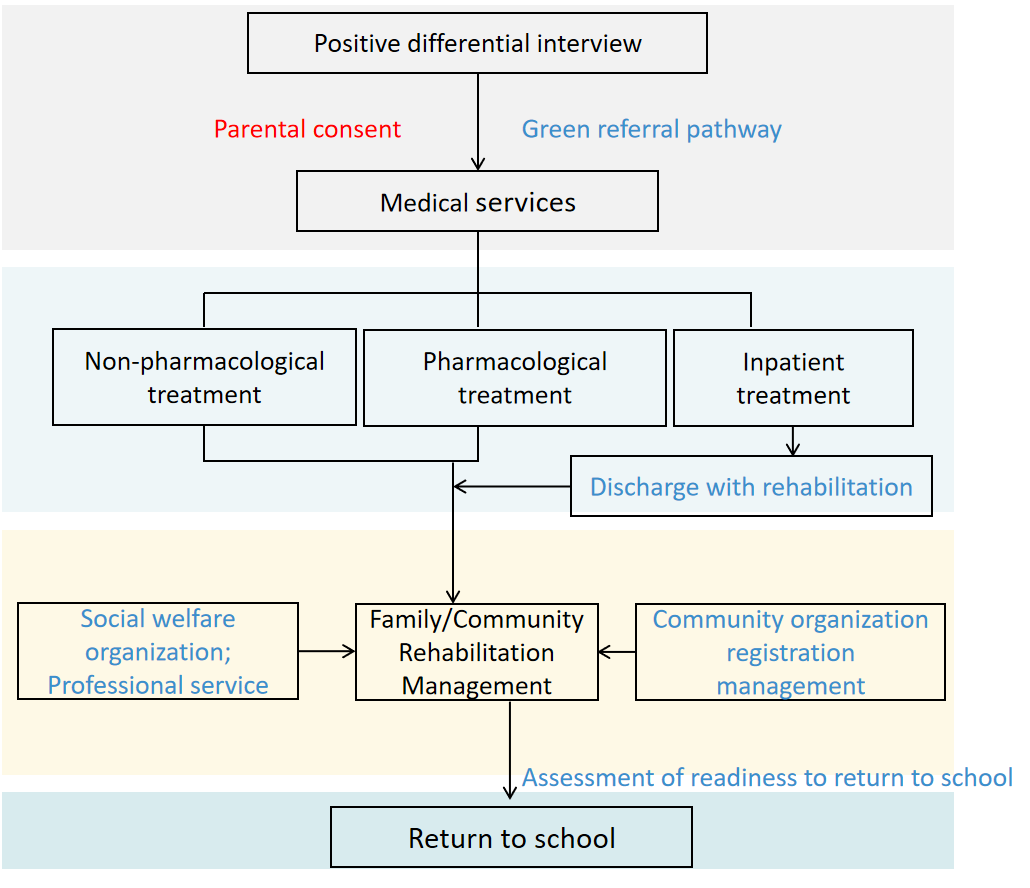


**4.2.4 Assessment of readiness to return to school**

Reentry Capability Assessment: The assessment work is carried out by clinical doctors, and the results are respectively provided to the school and parents.

Involvement of social welfare organizations in the assessment index system for the mental health service capabilities of minors: Through expert discussions, expert inquiries, and statistical analysis, the index system is established for the qualification review and access management of social organizations participating in services for minors.

**4.2.5 Screening of Social Organizations**

From the qualified social organizations, select local service-oriented social organizations based on the ratio of 1 per county/district, and establish the Social Public Welfare Organization Mental Health Service Work Alliance relying on the "Nanchong Mental Health Association", conducting 4 rounds of training and assessment for the core members, unifying and standardizing the service process.

**4.3 Care for Underprivileged Children/Adolescents**

Services that combine individual and group approaches

**4.3.1 Definition**

Children who are in survival, development, and safety difficulties due to their own and family reasons, and need the care and assistance of the government and society, including de facto unattended children/adolescents, “left-behind” children/adolescents, “single-parent” children/adolescents, children/adolescents in especially difficult circumstance (CEDC), and orphan.

**4.3.2 Work objectives**

Identify and address the mental health issues faced by vulnerable children at the family, community, and societal levels, enhance the mental health service capabilities of grassroots organizations, social welfare organizations, and community volunteers, and create a conducive living environment for the physical and mental well-being of vulnerable children.

**4.3.3 Work Object**

At the institutional level:

- Nanchong Special Education School, Nanchong First and Second Welfare Institutes

- In each county, select 3 mountain schools where left-behind children are more concentrated

At the individual level:

- In the early stage of psychological health screening, identified disadvantaged children based on basic information, as well as a list provided by homeroom teachers during school surveys. All lists are recorded in a work log and shared with the school's psychological counseling teachers.

**4.4 Training Work System**

Hierarchical training classification

**4.4.1 Trainees**

Administrative leadership, head teacher, subject teacher, and full-time or part-time mental health teacher.

**4.4.2 Training objectives**

Through categorized training, enhance the risk awareness of administrative leaders, the cognitive level of mental health among primary and secondary school teachers, the teaching abilities of mental health educators, and intervention skills for psychological issues, promoting a deep integration of mental health education with daily teaching activities.

**4.4.3 Overall Work Framework**


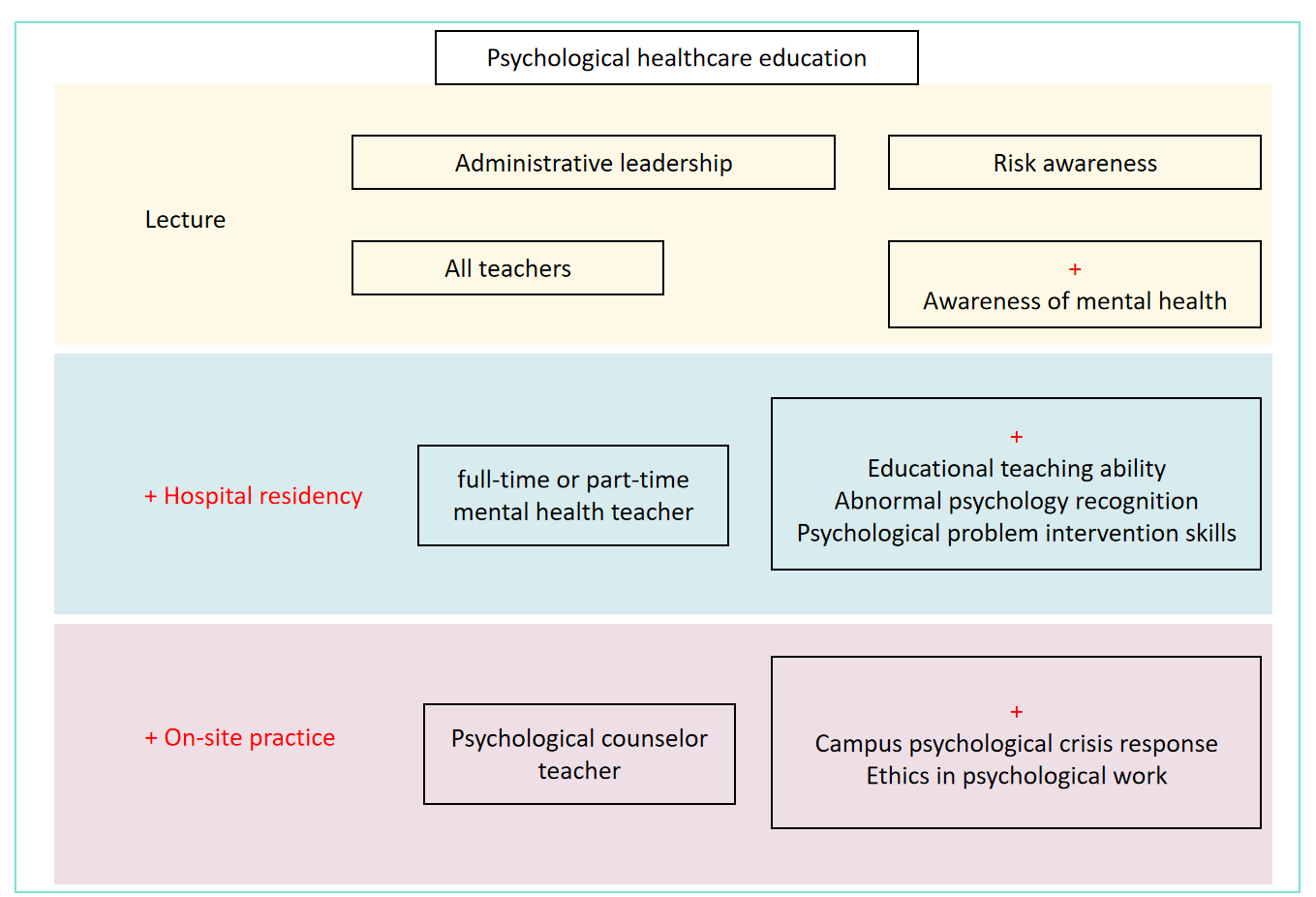


**4.4.4 Training methods**

Expert lectures: organized by county, combining online and offline methods.

Classroom demonstration teaching: organized by schools, using invitation system, with experts providing on-site training.

Hospital training: specialized training courses, open for registration, with 6 sessions per year, each session accommodating 10 teachers.

Intervention skill assistance: for 18 demonstration schools, training provided on-site by clinical psychologists.

**4.4.5 The training content (a total of 40 class hours)**

School Administrative Leadership (2 hours): School Mental Health Education Workflow.

Subject Teachers (12 hours): Developmental Psychology of Children and Adolescents, Positive Psychology, Interpersonal Communication and Emotional Management.

Head Teachers (16 hours): In addition to the subject teachers, include: Identification and Initial Handling of Common Psychological Issues in Students, Psychological Assistance Approaches, Parent-Child Relationships and Family Mental Health Education.

Full-time or part-time Mental Health Teacher (40 hours): In addition to the head teacher foundation, include: Introduction to School Mental Health Education, Psychological Assessment Techniques and Common Psychological Intervention Techniques, Campus Crisis Response System and Process Construction.

**4.4.6 Professional Development for full-time and part-time mental health teacher**

**4.4.6.1 Target population**

Full-time or part-time mental health teacher

**4.4.6.2 Schedule**

Every holiday period lasts for 7 days, with a total of 6 periods held each year.

**4.4.6.3 Educational objectives**

By attending further training in the clinical psychology department of the hospital under full-time teachers, one can master various professional techniques and skills in psychological counseling, correctly use psychological measurement tools, analyze and apply measurement results, provide guidance to students with psychological problems, accurately identify psychological disorders, and make timely referrals.

**4.4.6.4The content of further study (44 class hours)**

Basic skills in psychological counseling, intervention in psychological crises, identification and treatment of severe mental disorders, discriminative interviewing techniques, design and practice of group counseling, issues related to returning to school, ethics and laws, group counseling and individual case supervision.

**4.4.7 Assessment and Evaluation**

The assessment and evaluation of mental health education training adopt a combination of theory and practice, with assessment content set according to different levels of training. Grades are divided into excellent (not exceeding 20%), pass, and fail. Excellent and pass level mental health specialist and part-time teachers can receive a qualification certificate issued by the training center. After homeroom teachers and other teachers pass the training, their qualifications will be certified by the organizing unit.

**4.4.7.1 Assessment for full-time or part-time mental health teacher**

Theoretical Assessment (30% of the total score): The training center will prepare examination papers and answers based on the training content for a knowledge-based assessment. The assessment will cover basic theories and professional knowledge learned during the training.

Skills Assessment (30%): Conducted by the training center and course instructors.

Assessment content: (1) Scenario simulation: such as special topic guidance or lectures (segments); simulating organizing educational research activities; individual student counseling on specific issues; designing and conducting a mental health education class; case studies, etc. (2) On-site analysis: a segment of a class, a counseling case segment, a group counseling segment, a training or educational research activity, a typical teaching example, etc.

Practical Assessment (30%): Comprehensive evaluation by the training center, accounting for 30% of the total score. Assessment content: a professional teacher conducting a mental health education class or submitting a recording of a mental health education class.

Performance in Training (10%): Assessed by the training center, accounting for 10% of the total score. This includes attendance; active participation in training activities; compliance with training discipline and requirements, completion of assignments; outstanding self-study; strong simulation training ability, and active cooperation with course instructors.

**4.4.7.2 Assessment for head teacher**

Theoretical assessment (50%)

The training center will prepare the test papers and answers uniformly according to the training content to conduct knowledge-based assessments, accounting for 50% of the total score. The assessment content includes the basic theories and professional knowledge learned during the training.

Practical assessment (40%)

Comprehensively assessed by the training center. Assessment content: the case presented in the previous class meeting or the integration of mental health knowledge into educational activities.

Performance in training (10%)

Assessment records will be maintained by the organizing unit. This includes attendance; active participation in training activities; adherence to training discipline and requirements, completion of assignments; outstanding self-study; strong simulation training abilities, and active cooperation with teaching staff.

**4.4.7.3 Training on mental health education for all teachers**

The training center prepares exam papers and answers based on the training content for knowledge assessments, while the organizing unit is responsible for maintaining daily assessment records.

**4.5 Position Construction**

Incremental goal-oriented improvement plan

**4.5.1 The goal of building a school counseling center**

In the 9 counties (cities, districts) across the city, following a ratio of 2 schools per county (district), a preliminary plan has been made to establish 18 demonstration stations (points) for the mental health of minors.

**4.5.2 Construction and Evaluation**

**Recommendation for the Construction of School Counseling Rooms in Nanchong City**: Based on the guidelines and norms for the construction of primary and secondary school counseling rooms at the national and provincial levels, combined with the current situation of primary and secondary school counseling room construction in Nanchong City, a preliminary draft of the construction plan is discussed and formulated. After multiple discussions by internal project experts and commissioned external experts, the preliminary construction plan is repeatedly revised and finalized, and then submitted to the Civil Affairs Bureau and the Education and Sports Bureau of the city for review and approval.

**Evaluation Index System for the Service Capacity of School Counseling Rooms in Nanchong City**: Through expert discussions, expert inquiries, and practical testing, the evaluation index system for the service capacity of counseling rooms in primary and secondary schools is preliminarily established for the purpose of evaluating and providing improvement suggestions.

**4.5.3 Promotion of Demonstration Site Construction**

By determining phased goals through city-wide surveys, conducting on-site investigations of proposed construction sites reported from various areas to understand the current situation and needs, and establishing construction plans as follows:

Phase One: Drafting construction plans for counseling rooms.

Phase Two: Construction according to the drafted plans at each site。

Phase Three: Establishing counseling room evaluation standards to guide construction at each site.

Phase Four: Evaluation and acceptance, issuing certifications to sites that meet the evaluation standards.

Phase Five: Establishing stable connections with each site, providing professional support, and establishing a long-term mechanism for sustainable development.

**4.5.4 Trial of demonstration school listing system**

According to the "Service Capability Evaluation Index System of Psychological Counseling Rooms in Primary and Secondary Schools in Nanchong City," schools that have passed self-evaluation, initial evaluation, and re-evaluation will be listed by the "Zhengxin" project team.

Self-evaluation: Demonstration schools compare themselves to the "Evaluation Criteria for the Construction of Psychological Counseling Rooms in Primary and Secondary Schools in Nanchong City," and if they score above 60 points, they can apply for on-site initial evaluation by the field team.

Initial evaluation: After receiving the application, the field team organizes experts to visit the school, evaluate and score through on-site observation, data review, interviews, and other methods.

Re-evaluation: The field team reports the initial evaluation results to the Office of the Municipal Leading Group for the Protection of Minors. After receiving three or more initial evaluation results, the office organizes personnel for batch re-evaluation.

Awarding: Demonstration schools that pass the re-evaluation with a score of 60 points or above are awarded the title of "Guo Cheng You Xin, Nanchong Zhengxin Health Project Demonstration Site."

**4.5.5 Linkage Mechanism Construction**

Establish a service relationship between the school counseling room and the mental health hospital, with the mental health hospital providing technical guidance to the counseling room.

**Mechanism**: Establish online and offline green channels between schools/communities/institutions and specialized mental health hospitals.

**Target**: Individuals or groups identified by the school's psychological counseling room assessment as needing upgraded intervention and treatment.

**Channels**: Mental health platforms, hotlines, internet hospitals.

**Trigger Process**: Appearance of warning signals - report to the school psychologist - inform parents if initial intervention is ineffective - seek help through the green channel - provide support.

**4.6 Popular Science Work**

Integration of Online and Offline Popular Science Work Plan

**4.6.1 Work Objectives**

Through continuous, systematic, and professional popularization of mental health knowledge, promote the audience to fully understand the psychological characteristics and major mental health issues of minors at different stages, gradually master the basic skills of prevention and response.

**4.6.2 Target Audience**

Parents of students, teachers, personnel or volunteers working with minors.

**4.6.3 Popular Science Principles**

**Scientific**: The content is correct, does not involve academic or viewpoint controversies, contains no factual, expression, or judgment errors, and is based on reliable scientific evidence.

**Public Welfare**: Does not include any commercial information, does not promote information that contradicts the output and goals of health education.

**Applicability**: Comprehensive coverage of hot topics in mental health issues of public concern, content and dissemination forms are in line with the cultural level and acceptance capacity of the target audience.

**Respect**: Avoid biased information related to policies, ethnicity, gender, religion, culture, age, or race.

**Originality**: Avoid distortion, tampering with others' creations, plagiarism, or theft of others' works, and do not belong to adaptations, translations, annotations, or compilations of others' existing works.

**4.6.4 Online Popular Science Workflow**

**4.6.4.1 Evaluate Audience Needs**

Based on data collected during the previous work processes such as psychological health assessments of minors, teacher training, post-service for patients and children in distress, conduct statistical analysis. Combine specialized surveys, clinical work experience, and expert discussions to determine the cognitive abilities, cognitive levels, cognitive shortcomings, and main points of interest of the target audience, and establish the framework for the work content.

**4.6.4.2 Determine Communication Formats**

Communication Platforms: TikTok, WeChat Official Accounts, WeChat Video Accounts

Priority Order: Videos, comics, animations, popular science articles, and audio

Frequency of Communication: 2 works per week

Timing of Communication: Disseminate knowledge related to specific occasions such as new student enrollment, college entrance exams, major holidays, etc., in a targeted manner.

**4.6.4.3 Creation of Popular Science Works**

Overall Concept: Easy to understand, diverse formats, appealing to the audience

Work Team: Includes creative team, review team, publicity team, and management team

Content Writing: Elaborate on the theme by explaining why a certain phenomenon occurs, why things are done in a certain way, or specific instructions on how to do something.

**4.6.4.4 Work Review and Publication**

Adopt a three-level review system:

Initial review: Review the writing norms, focusing on (1) whether the information sources and evidence are cited, (2) whether the writing logic is clear, and if the content is relevant, (3) whether the article is original and eliminate articles with low innovation.

Professional review: Ensure the professionalism of popular science content to prevent social confusion, public panic, or mental and physical health damage caused by insufficiently scientific or ambiguous information expression.

Normative review: Ensure no conflicts with laws and regulations, social norms, ethical standards, or authoritative information to avoid negative public opinion.

**4.6.4.5 Dissemination Effect Evaluation**

Special surveys: Conduct individual and group interviews with parents, teachers, and volunteers once per quarter, and biannually conduct surveys to understand the main issues regarding the acceptability of content and dissemination methods, as well as the incentive effects on behavior change, serving as a basis for further optimization.

Online evaluation: Analyze the reading frequency, sharing frequency, completion rate, etc., of each work once per quarter to optimize dissemination channels and forms.

**4.6.5 Offline Popular Science Workflow**

**4.6.5.1 Establishing Popular Science Work Team**

Volunteer Team: Comprised of university students and social welfare organizations, responsible for the implementation of offline activities.

Planning Team: Consisting of frontline psychological teachers and psychiatrists, responsible for designing activity plans for different themes.

Expert Team: Comprised of experts in mental health education from universities and influential experts from social welfare organizations, responsible for ensuring the quality of activity plans.

Management Team: Selected from key members of the "Zhengxin" Health Project in Nanchong and the main members of the Nanchong Science Popularization Base, responsible for comprehensive management and service throughout the entire process of implementing offline activities.

**4.6.5.2 Popular Science Activities in Schools**

Utilize large-scale mental health-related festivals such as the "525 Mental Health Festival" or the "125 Mental Health Awareness Month" to conduct life education, interpersonal harmony, neuroscience, and other suitable popular science activities for different stages in primary, middle, and high schools. Use various forms such as carnivals, popular science knowledge Q&A sessions, and lectures to cultivate students' awareness of mental health and enhance their ability to maintain mental health, creating a better campus mental health atmosphere.

**4.6.5.3 Popular Science Activities in Communities**

Utilize major mental health-related holidays such as "World Mental Health Day" or "World Sleep Day" to spread popular science knowledge in areas where people gather in villages and urban communities using bulletin boards, LED screens, printed materials, and other methods to enhance citizens' mental health literacy, creating a social environment conducive to the physical and mental health development of minors.

**4.6.5.4 Activity Evaluation**

Special Surveys: Randomly select one remote rural school, one school in urban-rural fringe areas, and one urban school each year for personal interviews or group interviews with students, parents, teachers, volunteers, and school administrators. Conduct a questionnaire survey after the implementation of each new activity plan to understand the main issues existing in terms of the attractiveness, acceptability, and the effects on cognitive and attitude changes of the activity content, serving as a basis for further optimization.

**5. Project guarantee**

**5.1 Secretary Group Work**

Issue sorting feedback, upload and issue information

**5.1.1 Work Objectives**

Establish a comprehensive information platform, comprehensive coordination platform, comprehensive service platform to facilitate the flow of data and information among various groups, and promote the collaborative work of each working group.

**5.1.2 Work Content**

Build the "Pure Heart" Health Project information database, comprehensively collect, analyze, and provide feedback on project operations, regularly prepare work briefings, and provide feedback to various work groups and related departments.

Strengthen the coordination and linkage between the project management team and various work groups, establish a mechanism for issue feedback and resolution, and facilitate the flow of project operations.

Enhance coordination and connectivity, strengthen the forward-looking and anticipatory nature of work, provide high-quality and efficient services and decision-making basis for the management team, and provide information, data, and coordination services for each working group.

**5.2 Logistics Support Team Work**

**5.2.1 Work Objectives**

To improve the various rules and regulations of the logistics support team, enhance the service and support awareness of logistics support personnel, implement logistics management measures, strengthen project activity support and safety management, continuously improve project hardware and software construction, and ensure the steady and efficient operation of the "Healthy Heart" project.

**5.2.2 Job Responsibilities**

Assist the "Positive Heart" Health Project Team in formulating logistics support plans and safety management plans;

Assist the "Positive Heart" Health Project Team in internal management, providing timely and effective logistics support services for other work groups to carry out on-site activities;

Responsible for the management of office space and equipment for the "Positive Heart" Health Project, as well as activity safety management work;

Responsible for ensuring the various activities and meetings of the "Positive Heart" Health Project;

Complete relevant tasks assigned by the "Positive Heart" Health Project Team.

**6. Project Promotion**

**6.1 Work Objectives**

**6.1.1 Innovate the promotion mode to support the mental health of minors.**

Grasp the laws and characteristics of internet communication in the new era, empower technology to unleash the effectiveness of positive online promotion, and launch a series of key columns, quality reports, popular science comics, etc., around the themes of "positive mindset" and the "seven major actions". The promotional content should be able to integrate into the "youth group" and the "post-wave circle", telling stories in the "youth language" and "adolescent language", to "keep up" and "not be absent", and to generate empathy with the audience, guiding the healthy and robust growth of young people mentally.

**6.1.2 Tell a good story of "Zhengxin" and create a unique business card for Nanchong with heart.**

In-depth exploration of the connotation of the "Zhengxin" health project, profound refinement of classic cases, the improvement of content quality and innovative expression should complement each other, forming theme stories that resonate with the core values of Nanchong's development. Through official and self-media dissemination, embed "Zhengxin" into the urban brand image of Nanchong.

**6.1.3 Make a summary of the experience and form a replicable publicity model.**

Nanchong, as a pilot city for the "Zhengxin" Health Project, should scientifically formulate a plan, adhere to the idea of trying out, improving, and perfecting simultaneously, and do a good job of summarizing beneficial experiences and practices in publicity work to form a promotable publicity model.

**6.2 Forms of Publicity**

**6.2.1 Deeply cultivate traditional media to address profound societal topics.**

Organize special interviews, popularize knowledge on mental health, showcase project effectiveness, and report news in newspapers or on television, continuously follow up and report in depth.

**6.2.2 Leverage emerging media to attract social attention through traffic.**

Utilize the momentum of the new media matrix ("two microblogs and one short video platform"), regularly release event videos and reports on new media platforms, promote on major media websites, foster a positive interaction between traditional radio programs and new media, attract more audiences, expand influence, and spread the care and warmth of the municipal party committee and government for the mental health of minors even further.

6.2.3 Engage with community schools and various parties to proactively speak out. Move the publicity front line forward, collaborate with community schools as a whole, resonate on the same frequency, interact well with students, teachers, and parents, proactively share positive stories about the mental health of minors in Nanchong, and speak up for vulnerable groups.

6.2.4 Organize a series of activities to unite the forces of positive public welfare. Carefully plan public fundraising, charity assistance actions, and actively gather various charitable resources through cultural and sports activities, leverage public welfare forces, and promote the development of mental health among minors.
